# Deletions of chromosomal regulatory boundaries are associated with congenital disease

**DOI:** 10.1186/s13059-014-0423-1

**Published:** 2014-09-04

**Authors:** Jonas Ibn-Salem, Sebastian Köhler, Michael I Love, Ho-Ryun Chung, Ni Huang, Matthew E Hurles, Melissa Haendel, Nicole L Washington, Damian Smedley, Christopher J Mungall, Suzanna E Lewis, Claus-Eric Ott, Sebastian Bauer, Paul N Schofield, Stefan Mundlos, Malte Spielmann, Peter N Robinson

**Affiliations:** Department of Mathematics and Computer Science, Free University Berlin, Takustr. 9, Berlin, 14195 Germany; Max Planck Institute for Molecular Genetics, Ihnestr. 63–73, Berlin, 14195 Germany; Institute for Medical and Human Genetics, Charité-Universitätsmedizin Berlin, Augustenburger Platz 1, Berlin, 13353 Germany; International Max Planck Research School for Computational Biology and Scientific Computing, Ihnestr. 63–73, Berlin, 14195 Germany; Wellcome Trust Sanger Institute, Hinxton, CB10 1SA UK; Oregon Health & Science University, Department of Medical Informatics & Clinical Epidemiology, 97239, Portland, OR USA; Genomics Division, Lawrence Berkeley National Lab, 1 Cyclotron Rd., Berkeley, 94720 CA USA; The Jackson Laboratory, 04609, Bar Harbor, ME USA; University at Cambridge, Department of Physiology, Development and Neuroscience, Downing Street, Cambridge, CB2 3EG UK; Berlin-Brandenburg Center for Regenerative Therapies (BCRT), Augustenburger Platz 1, Berlin, 13353 Germany

## Abstract

**Background:**

Recent data from genome-wide chromosome conformation capture analysis indicate that the human genome is divided into conserved megabase-sized self-interacting regions called topological domains. These topological domains form the regulatory backbone of the genome and are separated by regulatory boundary elements or barriers. Copy-number variations can potentially alter the topological domain architecture by deleting or duplicating the barriers and thereby allowing enhancers from neighboring domains to ectopically activate genes causing misexpression and disease, a mutational mechanism that has recently been termed enhancer adoption.

**Results:**

We use the Human Phenotype Ontology database to relate the phenotypes of 922 deletion cases recorded in the DECIPHER database to monogenic diseases associated with genes in or adjacent to the deletions. We identify combinations of tissue-specific enhancers and genes adjacent to the deletion and associated with phenotypes in the corresponding tissue, whereby the phenotype matched that observed in the deletion. We compare this computationally with a gene-dosage pathomechanism that attempts to explain the deletion phenotype based on haploinsufficiency of genes located within the deletions. Up to 11.8% of the deletions could be best explained by enhancer adoption or a combination of enhancer adoption and gene-dosage effects.

**Conclusions:**

Our results suggest that enhancer adoption caused by deletions of regulatory boundaries may contribute to a substantial minority of copy-number variation phenotypes and should thus be taken into account in their medical interpretation.

**Electronic supplementary material:**

The online version of this article (doi:10.1186/s13059-014-0423-1) contains supplementary material, which is available to authorized users.

## Background

Genomic deletions and duplications result in the loss or gain of specific genomic segments and thus are referred to as copy-number variants (CNVs). The phenotypes of CNV disorders are often complex, commonly involving intellectual disability and multiple congenital anomalies [1]. The phenotypic abnormalities seen in some diseases associated with CNVs are thought to be related to altered gene dosage effects of one or more genes located within the CNV. For instance, Williams syndrome (WS) is a multisystem disorder that results from heterozygous deletion of 1.5 to 1.8 Mb on chromosome 7q11.23, which contains approximately 28 genes [2]. Some of the phenotypic abnormalities of WS have been attributed to hemizygosity of individual genes located within the deleted region. Thus, hemizygosity for the *ELN* gene is thought to cause the supravalvular aortic stenosis [4], *LIMK1* hemizygosity is implicated in the impaired visuospatial constructive cognition [3] and *GTF2I* hemizygosity is thought to contribute to the mental retardation in WS patients [5].

Alteration of gene dosage by deletion or duplication or by disruption of genes located at the boundaries of CNVs thus represents a plausible pathomechanism for many phenotypic abnormalities seen in CNV disorders. However, structural variations such as CNVs, inversions or translocations can also change the regulatory context of genes, thereby disturbing the delicate balance between enhancers, silencers and insulators by interfering with the complex chromosomal looping and interaction mechanisms of promoters and one or more cis-regulatory elements. These changes in the regulatory environment of genes can result in misexpression and subsequent deregulation of signaling [6-8].

Long-range looping interactions over tens or even hundreds of kilobases together with three-dimensional nuclear organization, involving the positioning of genes, regulatory sequences and DNA binding proteins, help determine which genes are transcribed at any given time [9,10]. Hi-C is a method that probes the three-dimensional architecture of whole genomes by coupling proximity-based ligation with massively parallel, next-generation sequencing [11]. Recently, Hi-C was used to identify megabase-sized local chromatin interaction regions termed ‘topological domains’; the domains represent highly self-interacting regions bounded by narrow segments where the chromatin interactions appear to end abruptly [12]. Topological domains were suggested to represent chromosomal units that serve to spatially accommodate enhancer–promoter interactions and control gene expression levels across cell populations [13]. The boundary regions between the domains are associated with CCCTC-binding factor (CTCF) binding sites, cohesin binding sites and active transcription of housekeeping genes [12]. Recent knock-down experiments suggest that CTCF and cohesin contribute differentially to chromatin organization and gene regulation, but surprisingly depletion of both was not accompanied by disruption of topological domain organization [14]. Therefore, it remains unclear whether the observed topological domains are the cause of genomic interaction or a consequence [15], but the boundaries between the domains might function as regulatory barriers by inhibiting the interaction of enhancers/silencers in one domain with genes in the adjacent domain [16]. Recent studies in *Drosophila* suggest that insulator proteins are frequently found at topological domain boundaries (TDBs) [17]. It was also shown that insulators can organize and support very long-range functional interactions between regulatory elements at distances of up to several megabases [18,19]. Since insulator proteins mediate not only enhancer blocking but also contribute to the organization of chromosome architecture and the integrity of regulatory elements, they have been dubbed architectural proteins [17]. The role of these architectural proteins in TDBs in vertebrates is currently being investigated.

We recently identified the etiology of Liebenberg syndrome, an autosomal-dominant upper-limb malformation, as a homeotic limb transformation in which the arms acquire morphological characteristics of a leg. We characterized deletions in the vicinity of *PITX1* in patients with Liebenberg syndrome. *PITX1* is a homeobox gene that plays a role in specifying the identity or structure of the lower limb. The structural changes are likely to remove a barrier element that separates the *PITX1* regulatory domain from neighboring regulators. In Liebenberg syndrome, a highly conserved non-coding enhancer element, hs1473, which is normally separated from *PITX1* by a TDB, was relocated into the vicinity of *PITX1*. Element hs1473 was shown to have forelimb-specific activity in mouse embryos, and transgenic hs1473-*Pitx1* mice showed features characteristic of *Pitx1* misexpression at embryonic day 15.5, as well as phenotypic features of forelimb-to-hindlimb transformation [20]. These observations suggested that the pathomechanism of Liebenberg syndrome can best be explained by a topological domain boundary disruption (TDBD) between an enhancer with activity in the forelimb and a gene that is phenotypically related to the clinical manifestations observed in individuals with Liebenberg syndrome [21]. We will refer to this phenomenon as ‘enhancer adoption’.

This observation motivated us to ask whether computational evidence can be obtained for additional CNVs with an analogous pathomechanism by searching for a bioinformatic signature suggestive of enhancer adoption. Here, we perform a systematic computational analysis of phenotypes of patients in the DECIPHER database [22]. Our results suggest that a substantial proportion of CNVs are associated with phenotypes that can be partially or completely explained by disruption of genomic barrier effects associated with ectopic activation of phenotypically relevant genes.

## Results and discussion

In this work, we present a computational analysis of the hypothesis that the disruption of TDB regions may contribute to or even be the major factor of the phenotypes observed in a subset of CNV disorders. We developed an analysis strategy that relates the phenotypic features of the CNV disorders to the locations of genes and TDBs within and near to the CNV as well as the phenotypic features of monogenic disorders affecting these genes.

Our approach involves comparing the phenotypic features associated with the CNVs with the phenotypic features associated with Mendelian diseases of single genes located within or adjacent to the CNVs. To do so, we perform semantic similarity analysis using the Human Phenotype Ontology (HPO) as described in detail in the Materials and methods. We define a gene as being *phenotypically relevant* if mutations in that gene lead to a Mendelian disease with phenotypic abnormalities that are similar to those of the CNV disorder (such as the genes *ELN*, *LIMK1* and *GTF2I* in WS, as described above). We analyzed 2,300 deletions in DECIPHER for which phenotype data were available, and found that the degree of similarity between CNV phenotypes and phenotypes associated with single genes located within the CNVs was significantly higher than for random deletions (19.6±28.8 compared to 14.2±25.6; *P*=8.54×10^−67^, Wilcoxon test). This result suggests that our computational approach of ‘explaining’ the phenotypic features of CNVs is applicable to the analysis of deletions in the DECIPHER database.

**Figure 1:**
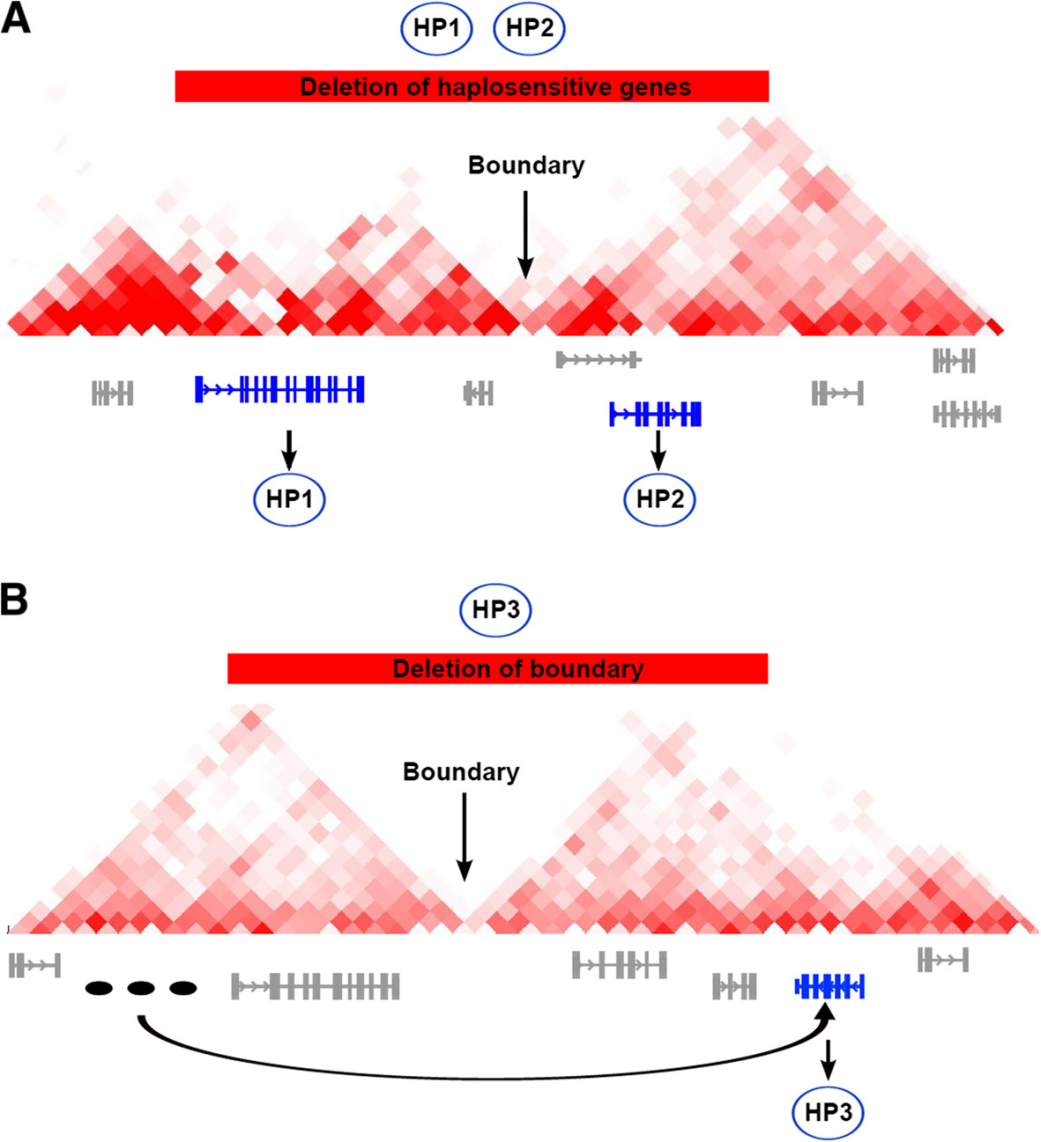
**Models of deletion pathomechanism.** In each panel, an exemplary deletion is shown as a red bar, a TDB is indicated with a black arrow, and genes associated with the phenotypes of the CNV patient are shown in blue, other genes in gray. Phenotypic abnormalities are represented as exemplary HPO terms (HP1, HP2 and HP3). Three tissue-specific enhancers are shown in (B) as black ovals. **(A)** Gene-dosage effect (GDE). A deletion leads to a reduction in the dosage of haplosensitive genes located within the CNV. The individual with the deletion has two phenotypic abnormalities (HP1, HP2) resulting from deletion of two haplosensitive genes. A Mendelian disease related to mutations in the first gene is associated with HP1, and a Mendelian disease related to mutations in the second gene is associated with HP2. **(B)** Topological domain boundary disruption (TDBD). Removal of the topological domain boundary allows the tissue-specific enhancer inappropriately to activate a phenotypically relevant gene located adjacent to the deletion, a phenomenon that we refer to as *enhancer adoption*. In this case, the individual with the deletion has a phenotypic abnormality (HP3) that is also seen in individuals with a Mendelian disease related to a mutation in the gene adjacent to the deletion.

We reasoned that deletions whose pathomechanism involves disruption of a TDB could be identified by searching for a specific bioinformatic signature whereby the deletion removes one or more TDBs and thereby brings a tissue-specific enhancer into the vicinity of a phenotypically relevant gene. On the other hand, CNVs whose pathomechanism primarily involves a gene dosage effect could be identified by the presence of one or more phenotypically relevant genes within the CNV without the presence of tissue-specific enhancers or relevant genes directly surrounding the CNV. In the following, we will refer to these categories as TDBD and gene-dosage effect (GDE) (Figure 1).

### Distribution of topological domain boundaries in pathogenic and neutral deletions

A total of 7,535 CNV cases from the DECIPHER database [22] were examined. The CNVs had an average size of 3.61 Mb, including 4,055 deletions, 2,300 of which were annotated with at least one HPO term. In this work, we concentrate on deletions. We first analyzed the relationship of the CNVs to the TDBs. There were a total of 3,026 non-overlapping boundaries in the human genome, encompassing a total of 134.32 Mb sequence and corresponding to roughly one boundary per million nucleotides of the haploid genome. Correspondingly, the CNVs contained 3.3 boundaries on average. We compared these figures to those obtained for a set of 1,958 deletions derived from adult probands investigated in genome-wide association studies by the Wellcome Trust Case Control Consortium 2 (WTCCC2), and which we will therefore regard as non-pathogenic control deletions in the context of congenital disease that is the focus of our analysis in this paper (Table 1).

**Table 1:** **CNV data from DECIPHER and control CNVs taken from the WTCCC2 study**

**Data**	***n***	**Length (Mb)**	**HPO terms**	**TDBs**	**Genes**
DECIPHER					
CNV cases	7,535	3.61 (±7.54)	3.3 (±4.9)	3.3 (±7.6)	29.2 (±53.6)
Deletions	4,055	3.68 (±5.74)	3.6 (±4.2)	3.4 (±5.6)	27.7 (±37.6)
Deletions with phenotype data	2,300	3.7 (±5.0)	5.6 (±4.7)	3.5 (±5.2)	27.3 (±32.7)
Deletions with unique target phenotype	922	4.6 (±5.3)	7.5 (±5.1)	4.3 (±5.7)	33.3 (±35.0)
WTCCC2 Controls					
Probands	5,919	0.428 (±0.29)	0.0 (±0.0)	0.099 (±0.37)	2.9 (±4.2)
Deletions	1,958	0.414 (±0.27)	0.0 (±0.0)	0.071 (±0.29)	2.3 (±2.9)

The mean value (± one standard deviation) is shown for the length of the CNV in megabases (Mb), the number of HPO terms used to annotate the CNV (only DECIPHER), as well as the number of TDBs and the number of genes contained within the CNV.

CNV, copy-number variation/variant; HPO, Human Phenotype Ontology; Mb, megabase; TDB, topological domain boundary; WTCCC2, Wellcome Trust Case Control Consortium 2.

**Figure 2:**
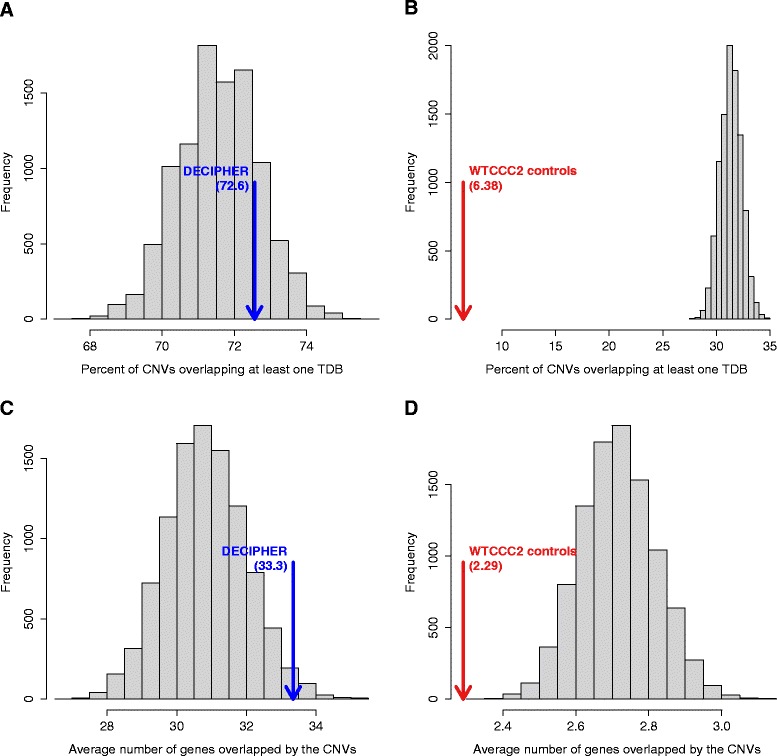
**Non-pathogenic deletions are depleted in genes and topological domain boundaries.** **(A,B)** Percentage of deletions that overlap at least one topological domain boundary (TDB). **(A)** DECIPHER and **(B)** WTCCC2. Deletions are compared with randomized data in which the same number of deletions (922 for DECIPHER and 1,958 for WTCCC2) of the same sizes as the original deletions were placed at random positions of the genome. This randomization was performed a total of 10,000 times, and the empirical distribution of TDB overlaps is plotted as gray bars. **(C,D)** Average number of genes overlapped by **(C)** DECIPHER and **(D)** WTCCC2 deletions compared with randomized data generated as in **(A)** and **(B)**. CNV, copy-number variation/variant; TDB, topological domain boundary; WTCCC2, Wellcome Trust Case Control Consortium 2.

Unsurprisingly, the mean size of the DECIPHER deletions was substantially higher than that of the control deletions (3.7±5.0 Mb vs 0.414±0.27 Mb). Of all 922 DECIPHER deletions analyzed, 72.6% overlap at least one TDB completely. This in itself is not significantly different from random expectation (71.6%) (Figure 2A). In contrast, 6.38% (125 of 1,958) of the non-pathogenic deletions overlap at least one boundary. We estimated the expectation by randomly placing equally sized deletions onto the genome and calculating the percentage with at least one overlapping topological domain boundary. We performed 10,000 simulations in which 1,958 deletions of the same sizes as the 1,958 original WTCCC2 deletions were placed at random positions of the genome, which displayed a mean of 31.3±1*%* deletions overlapping at least one TDB (Figure 2B). None of the randomized data sets have a lower or equal rate of boundary overlaps, corresponding to an empirical *P* value of *P*<10^−4^. Thus the benign control CNVs are significantly underrepresented at TDB regions. A similar analysis showed that WTCCC2 deletions overlap a lesser number of genes than would be expected by chance (Figure 2D) and the DECIPHER deletions overlap more genes than expected (Figure 2C).

We were therefore motivated to investigate how common TDBD is among pathological deletions associated with congenital anomalies. However, given that the mean size of the deletions in DECIPHER is 3.68 Mb, with over three TDBs being removed on average, the mere fact that a pathological deletion disrupts a TDB is not surprising. We therefore reasoned that it is necessary to take tissue specificity of enhancers as well as the phenotypic abnormalities associated with genes within and adjacent to deletions into account to assess the potential association of TDBD with deletion phenotypes.

### A computational phenotypic signature of topological domain boundary disruption

We reasoned that if TDBD is responsible for the pathogenesis of a sizable number of CNVs, then we should be able to detect a corresponding bioinformatic signature significantly more often than would be expected by random chance. To test this hypothesis, we developed a strategy for predicting computationally which CNVs are most likely to be partially or completely related to TDBD by comparing the phenotypes of the CNVs with the phenotypes of single-gene diseases of genes located within or adjacent to the CNVs and comparing their distribution with that of predicted tissue-specific enhancers (Additional file 1: Figure S2).

DNase-sequencing (DNase-seq) experiments from the National Institutes of Health’s Roadmap Epigenomics Mapping Consortium (NIH REMC) offer a unique resource for identifying enhancers. DNase I hypersensitivity, as measured by DNase-seq, has been used previously to characterize human cell lines, revealing cell-type-specific promoters and enhancers [23-25]. The human genome is thought to harbor at least 400,000 enhancers [26], many of which exhibit tissue or developmental-stage specificity [27].

**Figure 3:**
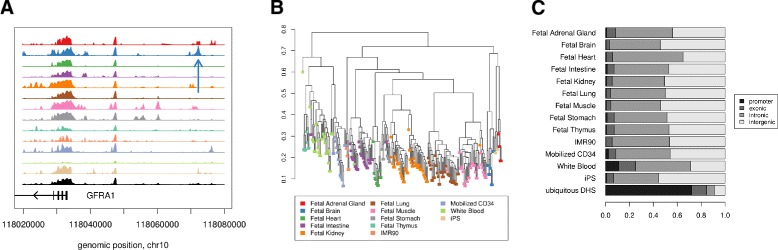
**DNase I hypersensitive sites (DHSs).** **(A)** Tissue-specific DHSs were predicted on the basis of DNase-seq data if chromatin accessibility was significantly higher in a given tissue than for the average over all cell types (ubiquitous DHS, black track). The vertical blue arrow indicates one of the top fetal-brain-specific DHSs more than 30 kb proximal to a promoter of *GFRA1*, a glial-cell-line-derived neurotrophic factor. Tissue types are color coded as in **(B)**. **(B)** Hierarchical clustering of samples by DNase-seq profiles. The distance between samples was defined as 1−cor(*x*,*y*). Correlations were calculated between the log counts of DNase-seq reads in 200-bp non-overlapping windows. **(C)** Locations of cell-type-specific DNase I hypersensitive sites (CTS-DHSs) and ubiquitous DHSs. The top ranked CTS-DHSs fall mostly in intronic and intergenic regions. The majority of the top ubiquitous DHSs are in promoters. chr10, chromosome 10; CTS-DHS, cell-type-specific DHS; DHS, DNase I hypersensitive site; iPS, induced pluripotent stem cell; kb, kilobase.

While cell-type-specific DNase I hypersensitive sites (CTS-DHSs) have been identified by the Roadmap consortium [28], this previous method did not attempt to account for within-cell-type variability, a critical step in our methodology for generating the ranking of sites that are consistently hypersensitive in a given tissue, relative to an average profile of all cell types. For this work, we analyzed nine fetal tissues, two non-fetal primary cell types and two cell lines to identify genomic regions with high degrees of chromatin accessibility that are most specific for certain tissues (see Materials and methods for details). For each cell type, we determined a set of high-confidence CTS-DHSs using reproducibility of the top-ranked sites across replicates. As the cell types of the tissues of interest all reached maxima of reproducibility for more than 20,000 sites, this led us to conclude that we could use the top 20,000 sites for each cell type as proxies for tissue-specific enhancers in the rest of the study (Figure 3, Additional file 1: Figure S1 and Additional file 1: Table S1).

To test the hypothesis that the TDBD pathomechanism is contributory for a subset of CNVs, we first assigned each CNV case to one of the general target terms that represent the ten tissues for which specific enhancers are available (Table 2) by identifying the HPO target term with a maximum similarity to the CNV phenotype terms. For brevity, we will refer to these HPO terms as ‘target phenotypes’.

**Table 2:** **Tissue-specific enhancers and corresponding HPO terms for ten tissue types**

**Tissue**	**HPO term name**	**Term ID**	**Descendant terms**	**Genes**	**Cases**
Fetal adrenal gland	Abnormality of the adrenal glands	HP:0000834	65	75	2 (0.217%)
Fetal brain	Abnormality of the forebrain	HP:0100547	213	640	276 (29.9%)
Fetal heart	Abnormality of the heart	HP:0001627	273	491	236 (25.6%)
Fetal intestine	Abnormality of the intestine	HP:0002242	121	260	17 (1.84%)
Fetal kidney	Abnormality of the kidney	HP:0000077	184	383	77 (8.35%)
Fetal lung	Abnormality of the lung	HP:0002088	149	529	9 (0.976%)
Fetal muscle	Abnormality of the musculature	HP:0003011	667	1079	291 (31.6%)
Fetal stomach	Abnormality of the stomach	HP:0002577	24	116	10 (1.08%)
Fetal thymus	Abnormality of the thymus	HP:0000777	9	26	0 (0.0%)
White blood cells	Abnormality of leukocytes	HP:0001881	195	256	4 (0.434%)

For each tissue type, a corresponding HPO term was chosen, and CNV cases were assigned to the HPO term if the term itself or any of its descendant terms was used to annotate the CNV in the DECIPHER database (See Materials and methods for details). The column ‘Genes’ shows the number of genes associated with monogenic diseases that display the corresponding feature in the main HPO database. The column ‘Cases’ shows the number of individuals in the 922 DECIPHER deletions investigated in this work that were annotated to have the HPO term in question. CNV, copy-number variation/variant; HPO, Human Phenotype Ontology.

In our analysis, we assigned deletions to the category TDBD if they completely overlapped a TDB and a tissue-specific enhancer and a phenotypically relevant gene were identified surrounding the deletion with the enhancer and the gene being on different sides of the deletion. A deletion was assigned to the category GDE if it contained one or more genes that were phenotypically relevant to the CNV, that is, for which the phenogram score (see Materials and methods) was above zero, with the additional condition that no computational evidence for TDBD was present. Finally, a deletion was assigned to the TDBD only category if the phenotypic similarity score of genes adjacent to the deletion was higher than for genes within the deletion. Note that a gene or enhancer was considered to be adjacent to the deletion if it was located between the deletion breakpoint and the distal end of the affected topological domain (Figure 1).

**Figure 4:**
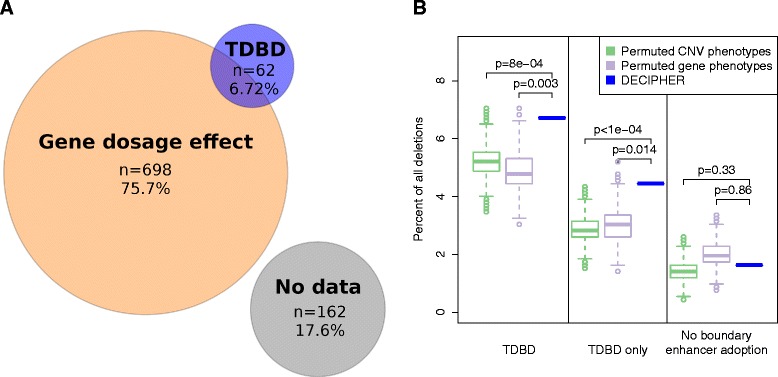
**Phenotype explanation of 922 CNVs as GDE or TDBD.** **(A)** Counts of DECIPHER deletions classified as GDE or TDBD. It was found that 41 of the TDBD cases did not demonstrate computational evidence of GDE and are indicated as TDBD only in **(B)**. **(B)** Proportion of CNVs predicted to correspond to TDBD, GDE or both, compared with randomized data by permutation of phenotype annotations of the DECIPHER patients (green) and permutations of phenotype associations of genes to monogenic diseases (purple). 6.72% of the deletions in the DECIPHER cases were predicted to be TDBD or mixed TDBD/GDE, compared to 5.18% on average for randomized (phenotype-shuffled) deletions (*P*=0.0008) and 4.88% for randomizations with permuted gene phenotypes (*P*=0.003). A pure TDBD mechanism was predicted for 4.45% of the DECIPHER cases and a mean of 2.84% of the randomizations with permuted CNV phenotypes (*P*<0.0001), and in 3.02% with permuted gene phenotypes (*P*=0.014). ‘No boundary enhancer adoption’ refers to deletions that do not overlap a boundary element but have a matching enhancer and gene signature in the 400-kb flanking regions. Here no significant enrichment over randomized data was observed, suggesting that the disruption of chromatin architecture contributes to TDBD-related enhancer adoption. CNV, copy-number variation/variant; GDE, gene-dosage effect; kb, kilobase; TDBD, TDB disruption.

In all, 4.45% of the CNVs from the DECIPHER dataset were assigned to the TDBD category, and an additional 2.28% were assigned to both TDBD and GDE (Figure 4A). Therefore, our results suggest that there may be a contribution of dysregulation of phenotypically relevant genes by disruption of TDBs in up to 6.72% of the DECIPHER deletions, compared to 75.7% with evidence only for GDE. Finally, for 17.6% of the cases, no phenotypic information for the genes within the deletions was available that matched the CNV phenotypes.

For comparison, we then performed an analysis of randomized data, whereby the deletion was assigned randomly to a different phenotypic category from Table 2. For instance, a deletion originally assigned to *Abnormality of the forebrain* might be assigned to *Abnormality of the kidney*. We then tried to identify the best ‘explanation’ for the random phenotype as GDE or TDBD as described above. Since the phenotypic spectrum of CNVs is complex and often multiple organs are affected, it is not surprising that some matches are found, but we reasoned that if the signal we observed for TDBD events in the real data was genuine, a lower proportion of random deletions would be placed into this category. In fact, there were significantly fewer deletions assigned to the category TDBD (*P*=8×10^−4^; Figure 4B). As an additional background model, we permuted the phenotype annotations of all human genes and found similar enrichment of TDBD deletions in the real data compared to randomized background (*P*=0.003; Figure 4B). The larger a deletion is, the more likely it is to contain haplosensitive genes whose deletion will cause a phenotype, whereas the chance that a deletion primarily acts by the TDBD mechanism should only depend on the enhancers and genes located adjacent to the deletion, and thus should not be dependent on the size of the deletion. Therefore, we investigated the relation between the number of topological domain boundaries affected by a CNV and the frequency of TDBD effect mechanisms. These data show that small deletions that overlap only one boundary show rates of 10% TDBD and thereby higher frequencies than larger deletions that overlap two or more domain boundaries. In all subsets of deletions that overlap up to three TDBs, the frequency of TDBD events was significantly higher in the DECIPHER CNV cases than in the randomized data with permuted CNV phenotypes (one TDBD: *P*=0.01; two TDBDs: *P*=0.0014; three TDBDs: *P*=0.0036; Additional file 1: Figure S3).

An alternative hypothesis to our concept of TDBD is simply that enhancer adoption occurs solely because a deletion brings a tissue-specific enhancer into the vicinity of a tissue-specific gene, regardless of chromosomal domains. The question boils down to whether TDBs tend to separate tissue-specific enhancers whose effect on phenotypically relevant genes would otherwise have a damaging effect. It would be difficult to provide a conclusive computational answer to this question for any specific CNV without extensive experimental validation. However, we did address the question by analyzing the 253 DECIPHER deletions that do not overlap any TDB. To do so, we searched in windows of 400 kb for the matching enhancer and gene signature on both sides of these deletions. We chose a distance of 400 kb because it corresponds to the median observed distance of 389.9 kb between CNV breakpoints and the next closest TDB (or in some cases the end of the chromosome or a region of unorganized chromatin at the border of a domain). Only 1.63% of the 922 DECIPHER deletions fulfilled our enhancer adoption criteria *without* overlapping a boundary element (Figure 4, right panel). This proportion is not more than expected from randomized data with permuted CNV phenotypes (1.44%, *P*=0.33) or permuted gene phenotypes (2.02%, *P*=0.86), which therefore suggests that the disruption of chromatin architecture by TDBD is a major factor in the enhancer adoption mechanism.

As a control for the specificity of enhancers, we repeated the TDBD analysis with the ubiquitous DHS and observed lower rates of TDBD events compared to the analysis with tissue-specific enhancers (3.69% for ubiquitous vs 4.45% for tissue-specific enhancers; Additional file 1: Figure S4A). Furthermore, the phenotypic similarity of genes adjacent to the deletion to the phenotypes of the patient was significantly higher for TDBD with tissue-specific enhancers compared to the ubiquitous enhancers (*P*=0.013; Additional file 1: Figure S4B).

### Model organism data increases the number of interpretable copy-number variants

We recently presented an ontology-based approach to measure similarities between human disease manifestations and the mutational phenotypes in model organisms to identify candidate genes located within CNVs that best explain the individual phenotypic features of the CNV [29]. Since there are considerably more mouse and zebrafish mutants with monogenic defects than the number of currently characterized Mendelian diseases of humans [30], we asked whether cross-species analysis would increase the percentage of CNVs that could be classified with our algorithm. As in our analysis of purely human disease data, we compared the similarity of the 2,300 DECIPHER deletion phenotypes to the phenotypes of the single-gene disorders of the genes located within the CNVs. However, here, we used the cross-species ontology Uberpheno [31] to exploit mouse and zebrafish annotations for these genes. The phenotypic similarity for the DECIPHER deletions was significantly higher than for randomized deletions (62.2±81.8 compared to 45.6±66.8; *P*=2.36×10^−58^). Using the cross-species data, we again analyzed the 922 DECIPHER deletions that had been assigned to a target phenotype corresponding to a tissue-specific enhancer. Compared with the purely human data, about 10% more cases could be classified for a total of 92% of all CNVs for which our phenotypic analysis allowed assignment to one of the categories TDBD and GDE. Compared to the rate of 4.45% TDBD events predicted with human data, 5.75% of deletions were characterized as purely TDBD using the model organism data. This was significantly more than for randomized data with permuted CNV phenotypes (*P*=0.011) and permuted gene phenotypes (*P*<0.001; Additional file 1: Figure S5).

### DECIPHER deletions with predicted TDBD pathomechanism

**Figure 5:**
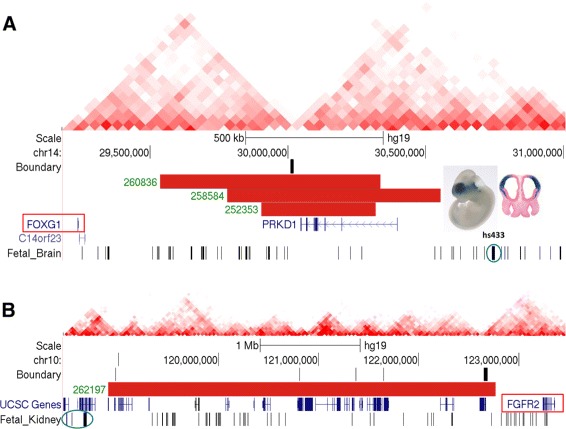
**DECIPHER CNVs whose pathomechanism can be explained by TDBD.** **(A)** Candidate TDBD at the *FOXG1* locus. Three of the 40 candidate TDBD cases identified in this study are located adjacent to the *FOXG1* gene. They are truncating mutations associated with neurodevelopmental phenotypes such as Rett syndrome [32]. All these microdeletions overlap the gene *PRKD1* and a topological boundary region but not *FOXG1* itself. Human element hs433 is shown as an image from the VISTA enhancer browser [37]. **(B)** A deletion of about 3.9 Mb on chromosome 10 leads to haploinsufficiency of a number of genes with no known phenotypic relevance to the CNV phenotype of *multiple renal cysts* (HP:0005562). The deletion also removes a total of five TDBs that lie between several predicted kidney-specific enhancer elements and the gene *FGFR2*. chr14, chromosome 14; CNV, copy-number variation/variant; kb, kilobase; Mb, megabase; TDB, topological domain boundary; TDBD, TDB disruption; UCSC: University of California, Santa Cruz Genome Browser.

We identified three patients with TDBDs at the *FOXG1* locus (Figure 5A). Mutations in *FOXG1* itself cause a congenital variant of Rett syndrome [32,33]. The patients with a TDBD at the *FOXG1* locus show severe Rett-like phenotypes similar to patients carrying *FOXG1* mutations. A recent study [34] showed misregulation of *FOXG1* in cell lines derived from patients with such deletions and proposed misregulation of *FOXG1* rather than haploinsufficiency of the gene contained within the deletion (*PRKD1*) as the primary pathomechanism. The authors suggested that a cis-acting regulatory sequence located in the deleted region more than 6 kb away from *FOXG1* might act as a silencer element at the transcriptional level. Our data, however, suggest that the deletions remove TDBs and bring ectopic fetal brain enhancers into the regulatory landscape of *FOXG1*. As shown in Figure 5A, several brain enhancers, including the enhancer element hs433, are located close to the breakpoint and are now free to act on *FOXG1* to cause misexpression in the brain of the affected individuals [35]. This mutation mechanism has also been described as enhancer adoption [36] and transgenic studies show that individual enhancer elements cloned in front of their ectopic target genes are able to recapitulate disease phenotypes in mice [20]. Therefore, we suggest that misexpression of *FOXG1* in the patients with the congenital variant of Rett syndrome can be better explained by TDBD than by a deletion of a silencer element.

A deletion of about 3.9 Mb on chromosome 10 leads to haploinsufficiency of a number of genes with no known phenotypic relevance to the CNV phenotype of *multiple renal cysts* (HP:0005562). The deletion also removes a total of five TDBs that lie between a predicted kidney-specific enhancer at chr10:118,480,800 to 118,481,000 and the gene *FGFR2*. Many fibroblast growth factors (FGF) and all of their receptors (FGFR) are expressed in the developing kidney, and overexpression of basic fibroblast growth factor in developing rodent kidneys can induce the formation of renal cysts *in vivo* [38]. In humans, activating and loss-of-function mutations in FGFRs cause syndromes that are sometimes associated with urogenital anomalies [39], including lacrimo-auriculo-dento-digital syndrome and Antley–Bixler syndrome, both of which can be caused by *FGFR2* mutations and in some cases are associated with severe congenital renal anomalies [40,41]. Therefore, we hypothesize that disruption of the TDBs in the deletion in DECIPHER case 262197 results in overexpression of *FGFR2* in the developing kidney with resultant formation of renal cysts (Figure 5B).

Two additional cases (not shown in Figure 5) showed deletions in the vicinity of the *DUX4* gene. Facioscapulohumeral muscular dystrophy is an autosomal dominant disease associated with reduction in the copy number of the D4Z4 repeat at chromosome 4q35. The reduction in D4Z4 copy number leads to reduced polycomb silencing and production of a chromatin-associated non-coding RNA that coordinates derepression of 4q35 genes including the transcription factor *DUX4* [42]. The resulting misexpression of *DUX4* in skeletal muscle may be associated with apoptosis of muscle cells [43,44]. A similar D4Z4 repeat array, which contains a paralog of *DUX4* at chr10:135,480,558 to 135,485,241, has been identified on chromosome 10q26, but contractions at the 10q26 locus are not pathogenic. DECIPHER case 249776 represents a deletion of chr10:130,955,710 to 135,397,841. The deletion removes two TDBs thereby bringing 107 muscle-specific enhancers into the vicinity of the chromosome 10 *DUX4* paralog. Similarly, DECIPHER case 4069 represents a deletion at chr10:129,690,073 to 135,422,505, which removes three TDBs and brings 33 muscle-specific enhancers into the vicinity of the chromosome 10 *DUX4* paralog. Both DECIPHER cases are associated with a number of features including *muscular hypotonia*, which was the feature leading to the characterization of the deletion as TDBD. Therefore, one possibility for the pathogenesis of this feature might be an inappropriate activation of the chromosome 10 *DUX4* gene by adoption of the muscle-specific enhancers.

Additional file 1: Table S2 provides an overview of the 41 DECIPHER CNVs classified as purely TDBD by our algorithm.

## Conclusions

In this work, we have provided suggestive computational evidence that a TDBD pathomechanism may be involved in a substantial minority of deletions recorded in the DECIPHER database. For the great majority of deletions and other CNVs identified to date, medical interpretation (‘explanation’ of the phenotypic features found in an individual with the CNV) has been based on a guilt-by-association approach, in which one compares the CNV phenotypic features with those associated with monogenic diseases of the genes located within the CNV. Thus, the explanation of the phenotypic feature supravalvular aortic stenosis in WS is thought to be haploinsufficiency of the elastin gene, because individuals with loss-of-function mutations in this gene have the identical phenotypic abnormality. Comprehensive experimental investigation of the pathomechanism of a CNV disease such as WS might involve the generation of mouse models in which the orthologous chromosomal regions have been removed but each of the genes in turn is ‘rescued’ by addition of a corresponding transgene construct. Since strategies such as this are currently unthinkable for investigating the pathogenesis of human CNV diseases, numerous computational approaches have been applied to investigate the pathogenesis of CNVs [29,45-48]. In the current work, we have shown that a computational approach to analyze deletions in light of adjacent tissue-specific enhancers and genes identifies up to around 10% of deletions in DECIPHER as having a potential contribution of the TDBD pathomechanism. While our approach does not provide proof of this pathomechanism, previous guilt-by-association approaches did not do so either. Our results do suggest that TDBD should be taken into account in the interpretation of deletions, and that corresponding experimental analysis of deletions may be fruitful for future research.

A limitation of our study is the fact that the size of deletions in DECIPHER (mean 3.68 Mb) is much greater than the mean distance between adjacent TDBs. In contrast, the deletions we identified in two individuals with Liebenberg syndrome were only 134 kb and 107 kb in size [20]. The larger deletions that are common in DECIPHER are more likely to have a complex mode of pathogenesis resulting from haploinsufficiency of one or even multiple genes located within the deletion and in some cases at least from the enhancer adoption mechanism [21]. However, we speculate that there may be a bias to submit cases with large CNVs to databases such as DECIPHER, because previous paradigms of CNV interpretation focused on a potential phenotypic relevance of genes located within the CNV itself, not on adjacent genes [49]. Therefore, it may be fruitful for future research to search specifically for smaller deletions that conform to the enhancer adoption pathomechanism described here.

We did not analyze duplications in our study. The location of duplicated copy can be adjacent to the original (tandem) or somewhere else in the genome, and a tandem duplication can be in the original orientation or inverted. Array CGH, which was used to generate the data investigated in our study, is not able to distinguish between these possibilities, each of which would be predicted to have a different effect on gene regulation by disruption of TDBs. However, a duplication could in principle bring elements that are normally separated by one or more TDBs into the vicinity of one another and thereby cause disease.

Our results have important implications for the medical and scientific interpretation of CNVs, and suggest that the pathomechanism of a sizable minority – up to even 11.8% – of CNVs may be related to the disruption of TDBs with misregulation of phenotypically relevant genes due to enhancer adoption. Currently, medical interpretation of rare CNVs often involves comparison of the phenotype seen in the patient with the CNV with that of monogenic diseases associated with genes located within the CNV. Our results suggest that it is also important to examine the topological domain structure in the region of the CNVs for the presence of tissue-specific enhancers and phenotypically relevant genes that lie adjacent to the CNV itself. It will also be important to develop experimental strategies for investigating these cases based on chromosomal conformation capture or similar approaches. Finally, the analysis described in this paper was made possible because of data shared by many in the community within the framework of the DECIPHER database, demonstrating the value of sharing genotype and phenotype information with appropriate data access conditions. Phenotypic data will continue to be key to understanding the medical relevance of genomic variation.

## Materials and methods

### Clinical and molecular copy-number variant data

The DECIPHER database is an online repository of rare genomic CNVs and associated phenotypic data [22]. For each of the 7,535 cases in DECIPHER, we considered only the single largest CNV, of which 4,055 were deletions. Of these, 2,300 were annotated with phenotypic data and were used for our analysis. We additionally compiled a set of CNVs from 5,919 individuals participating in WTCCC2 as common controls as previously described [45]. After mapping the genomic coordinates to the hg19 reference genome using the UCSC liftover tool [50], we again took only the largest CNV per case into account and analyzed only deletions. Our underlying assumption with this data is that CNVs observed among adults recruited as controls for genome-wide association studies are unlikely to be causative of congenital anomalies.

### Tissue-specific enhancer prediction

DNase-seq is a high-throughput experimental technology, which has been shown to be effective in identifying open chromatin regions that correspond to active gene regulatory elements. Nucleosome-depleted regions representing open chromatin are distinguished from DNA regions that are tightly wrapped in nucleosomes or in higher-order structures by the ability of DNase I to digest the sequences. DNase-seq identifies such DHSs by capturing DNase-digested fragments and sequencing them by next-generation sequencing [51]. Transcription factor binding is highly cell-type specific, and the investigation of differential DNase I hypersensitivity provides a general approach for predicting cell-type specific binding profiles [52]. In this work, we have developed a computational methodology to predict tissue-specific enhancers on the basis of differential DNase I hypersensitivity profiles from ten human tissues (Table 2). Accessible chromatin regions are preferentially cleaved by endonucleases, such as DNase I, and are therefore referred to as hypersensitive, and can be measured using DNase-seq by digesting chromatin with the endonuclease DNase I followed by next-generation sequencing. DNase-seq thus generates a genome-wide map of DHSs that reflects the degree to which sequence regions were accessible [53].

DNase-seq reads from the NIH REMC [54] were counted in 200-bp windows covering the human genome. Windows that overlapped repetitive elements in RepeatMasker with scores higher than 1,000 were eliminated leaving 9.7 million windows. Genomic range manipulation and counting were performed using BEDTools [55]. The logs of the counts plus a pseudocount of one were normalized for sequencing depth by multiplying each sample by the average read count over all samples divided by the sample’s average read count. For each sample, we counted the number of DNase-seq reads falling into non-overlapping 200-bp windows along the human genome excluding strong repeat sequences. After accounting for different sequencing depth in the samples, we generated an average profile for each tissue as well as for all tissues combined (ubiquitous DHS) (Figure 3). Using correlation to measure distance between DNase profiles, we were able to group samples by cell type with hierarchical clustering (Figure 3B). The differences for each 200-bp window and each tissue from the average profile were calculated and weighted by the pooled within-tissue standard deviation. This derived quantity corresponds to a *t*-statistic and measures the specificity of a DHS for the corresponding tissue. We then ranked all the 200-bp windows for each tissue such that top-ranked sites corresponded to the largest positive *t*-statistics.

We have shown that our quantitative measure of tissue specificity allows us to define a reproducible set of ranked DHSs. Next, we tested whether the location and the chromatin environment of the identified CTS-DHSs support our claim that the identified CTS-DHSs are indeed specific for a tissue or cell type. The top CTS-DHSs are located primarily in intronic and intergenic regions. This is in stark contrast to the top ubiquitous DHSs, of which 72% overlap promoter regions (Figure 3). These findings suggest that the CTS-DHSs are mainly enhancers, which may regulate nearby genes – a conclusion that has also been drawn in earlier studies about cell lines [23-25].

We used the profiles of the normalized log counts from each DNase-seq sample to find regions of similarity and difference across the tissue types. We created an average profile of DNase accessibility for each tissue type as well as across all tissue types (ubiquitous DNase hypersensitive sites). We then predicted tissue specificity based on a calculation of the within-tissue-type variance of DNase accessibility.

For a given window, let *X*_*i*_ be the log read count for sample *i*∈{1,…,*n*}. We denote the set of all samples belonging to a given tissue type *j*∈{1,…,*m*} as *C*_*j*_, i.e., *C*_*j*_⊆{1,…,*n*}. Note that in our work we assume that each sample *i* belongs to exactly one tissue type, that is, the *C*_*j*_ are pairwise disjunct. If we denote the the cardinality of *C*_*j*_ as *n*_*j*_, the average log read count for sample *j* is $\overline {X}_{j} = \frac {1}{n_{j}}\sum _{i \in C_{j}}X_{i}$, and thus the average log read count for the ubiquitous DHS is $X = \frac {1}{m}\sum _{j=1}^{m}\overline {X}_{j}$. Furthermore, the unbiased tissue-type variance is given by ${s_{j}^{2}} = \frac {1}{n_{j} - 1}\sum _{i\in C_{j}}(X_{i} - \overline {X}_{j})^{2}$. Assuming equal variance among tissue types, we derive for the pooled within-tissue-type standard deviation:

(1) $$ s = \sqrt{\frac{\sum_{j=1}^{m} (n_{j}-1) {s_{j}^{2}}}{\sum_{j=1}^{m} (n_{j}-1)}} = \sqrt{\frac{\sum_{j=1}^{m}\sum_{i \in C_{j}}\left(X_{i}-\overline{X}_{j}\right)^{2}}{\sum_{j=1}^{m} (n_{j}-1)}}.  $$

For tissue type *j*, the *t*-statistic is calculated as:

(2) $$ t_{j} = \frac{\overline{X_{j}}-\overline{X}} {\sqrt{1/m + 1/n_{j}}\cdot (s+s_{0})},  $$

where *s*_0_ is the mean of *s* over all windows to prevent division by small within-cell-type variance estimates [56]. The ranking of these *t*-statistics over all windows was used to quantify the cell-type specificity. Statistical analysis was carried out using the R statistics environment, using the sparse matrix package Matrix.

To estimate the number of reproducible top-ranked DHSs, all DNase-seq samples were split into two equally stratified groups. Then, within-cell-type standard deviations and CTS-DHSs were calculated separately for each group. For the top *n* sites, the reproducible ratio (the proportion of top CTS-DHSs that are shared between the two groups) was calculated. Looking at reproducible ranks (correspondence at the top plots) helps to determine at what cutoff the ranks transition from consistent ones into lower ranks dominated by noise [57,58]. Maxima were defined using interpolation of reproducible ratio curves (Additional file 1: Figure S1 and Additional file 1: Table S1).

### Topological domains and boundaries

Topological domain data from genome-wide higher-order chromatin interaction data in human embryonic stem cells [12] were downloaded [59] and mapped to hg19 coordinates using the UCSC liftover tool [50]. TDBs are defined as regions with size up to 400,000 bp (400 kb) between topological domain regions.

### Analyzing phenotypic similarity: human phenotype ontology and the Uberpheno ontology

The data for the analyzed CNV patients in the DECIPHER database are annotated with a set of phenotype terms from HPO. For each HPO term *t*, the information content *IC*(*t*) is calculated as the negative logarithm of the frequency of annotations to the term [60]:

(3) $$ \textit{IC}(t) = -\log p_{t},  $$

where *p*_*t*_ is the observed frequency of patients annotated to term *t* among all annotated patients in DECIPHER, $p_{t} = \frac {\text {patients with term}\,\,t}{\text {all patients}}$. Note that the annotation propagation rule applies here [61], i.e., if a patient is annotated to a term *t* then the patient is also annotated to all of the more general terms.

For some of the analyses described in this work, we assigned patients to one of ten phenotypic categories corresponding to the ten tissue-specific enhancers. This strategy was based on observations in families with *PITX1* mutations and for Liebenberg syndrome. The transcription factor *Pitx1* is expressed predominantly in the developing hindlimb and is only minimally expressed in the forelimb [62], suggesting that *Pitx1* is an important regulator of hindlimb identity. Both a missense mutation in the highly conserved homeodomain of *PITX1* as well as a 241-kb chromosome 5q31 microdeletion have been shown to result in clubfoot in humans [63,64], allowing *PITX1* to be assigned to the top-level category of genes with phenotypic relevance for the skeleton. In our previous work, we showed that heterotopic activation of *Pitx1* by tissue-specific skeletal (forelimb) enhancers leads to Liebenberg syndrome [20,21]. We note that the phenotypic features of these diseases are distinct (clubfoot with *PITX1* mutations and an upper-limb malformation in Liebenberg syndrome), but that they both affect the skeletal system. Therefore, we reasoned that if heterotopic activation of a gene by a tissue-specific enhancer is responsible for a CNV phenotype, then we should expect a phenotypic abnormality in the same organ system rather than necessarily an exact phenotypic match.

Therefore, we let *T*={*T*_1_,*T*_2_,…,*T*_10_} represent the ten HPO terms shown in Table 2, $\text {annot}_{j} = \left \lbrace t_{j_{1}}, t_{j_{2}},\ldots, t_{j_{m}}\right \rbrace $ be the *m* terms to which patient *j* is directly annotated, and desc(*T*_*i*_) represent all terms that are more specific descendants of term *T*_*i*_ as well as the term *T*_*i*_ itself. With *S*_*ij*_=desc(*T*_*i*_)∩annot_*j*_, patient *j* was assigned to term *T*^*j*^∈*T* by:

(4) $$ T^{j} = \mathop{argmax}\limits_{T_{i} \in T} \sum_{t\in S_{ij}} IC(t).   $$

We only included cases in the further analysis if they had at least one term in *S*_*ij*_ and for which there was a unique maximum for one of the ten *T*_*i*_. Then 922 of the 2,300 deletion cases could be assigned to one of the ten phenotype categories in Table 2 in this fashion. The remaining cases could not be classified because they did not share phenotype terms with any of the target terms (*n*=1,377). One case was excluded from further analysis because maximal values were obtained for more than one target term by Equation 4.

### Quantification of phenotypic similarities

The genomic coordinates of human genes in hg19 were retrieved from the UCSC known-genes table and mapped to Entrez Gene IDs. For the resulting 23,459 genes, only the longest transcript was considered. The similarity between the set of phenotype terms annot_*j*_ used to annotate a patient *j* and the set of terms associated with genes in the genomic region within or adjacent to a deletion is calculated as described previously [29] with some modifications. For each gene *g* in a region *G*_*CNV*_ within or adjacent of a deletion, a phenomatch score *S*_*g*_ is defined based on the information content of the term. For these calculations, the frequencies *p*_*t*_ were calculated based on HPO project annotations for human diseases [65]. For cross-species analyses, the frequencies *p*_*t*_ were calculated based on annotations to term *t* amongst all annotated genes in humans, mice and zebrafish in the cross-species phenotype ontology Uberpheno [31].

We define anc (*T*) as a function that for a given term or set of terms, returns the set of ancestral terms. The set of common ancestors of term *t*_*g*_ associated with gene *g* (*t*_*g*_) and the set of terms associated with the deletion observed in DECIPHER patient *j* (annot_*j*_) is defined as

(5) $$ \text{CA}(t_{g},\text{annot}_{j}) = \text{anc}(\text{annot}_{j}) \cap \text{anc}(t_{g}).   $$

We can now define the phenotypic similarity of an individual gene to the phenotypic abnormalities of the CNV as

(6) $$ S_{g}(g, \text{annot}_{j}) = \sum_{t_{g} \in T_{g}} \max \{IC(t) | t \in \text{CA}(t_{g},\text{annot}_{j})\}.   $$

Finally, the full phenogram score across all genes located within the CNV is calculated as the maximum of the phenomatch scores *S*_*g*_ of all genes within the CNV:

(7) $$ S_{PG}(G_{cnv}, \text{annot}_{j}) = \max_{g \in G_{cnv}} S_{g}(g, \text{annot}_{j}).   $$

An analogous score is calculated for the genes that are adjacent to the CNV:

(8) $$ S_{PG}(G_{\text{Adj}}, \text{annot}_{j}) = \max_{g \in G_{\text{Adj}}} S_{g}(g, \text{annot}_{j}).   $$

We note that in our previous work [29], we used a scoring scheme designed to identify all genes within the CNVs that were good candidates for contributing to the phenotypic spectrum of the CNV. This was possible because of our detailed manual biocuration of the 27 CNV syndromes. For the current project, we chose a scoring system that would look for a single gene within or adjacent to the CNV with the maximal phenotypic similarity, since the depth of annotations in DECIPHER is much less.

### Statistical analysis

To test whether the phenogram score in Equation 7 captures clinical similarities between deletions and the genes located within them (as with the *ELN* gene and WS as explained in the introduction), we placed each of the 2,300 DECIPHER deletions with at least one HPO term 100 times randomly on the genome and compared the distribution of phenogram scores of genes within the random deletions against those of the DECIPHER deletions with a Wilcoxon/Mann–Whitney test.

For a given patient assigned to the phenotype target term *T*, we define a deletion as TBDB, if it completely overlaps a TDB, has a *T*-specific enhancer in one region adjacent to the CNV and has a gene associated with *T* in the adjacent region located on the other side of the CNV. Adjacent regions span the genomic sequence from each end of the deletion up to the end of the current domain (Figure 1B).

To assess the statistical significance of TBDB events in DECIPHER, we simulated a background distribution by permuting the phenotype annotations in the following way. We assigned to each DECIPHER patient *i* the phenotype annotation of a randomly chosen DECIPHER patient *j* that is not in the same target term group as the original patient *i*. We repeated this procedure for all 922 deletion patients 10,000 times and computed the empirical *P* value as the fraction of randomizations for which a higher or equal rate of TBDB events as in the original annotation assignment is observed. As a further control, we permute the phenotype annotations not of the CNV patients but of the genes. To do so, we shuffled all 23,459 human gene IDs randomly and replaced each gene in the HPO annotation files with a random other gene. This approach to permutation holds the number of disease genes and depth of annotation constant. We computed an empirical *P* value as the proportion of 1,000 permutations in which a higher or equal rate of TBDB events was observed compared with the non-permuted gene phenotypes.

### Data and code deposition

Python scripts that implement the algorithms described in this manuscript have been deposited in GitHub [66]. This repository also contains files with data on the tissue-specific enhancers used in this analysis. Additionally, source code for performing simple statistics on sparse data sets without losing sparsity that was used for the analysis of tissue-specific enhancers has been deposited as SparseData in GitHub [67]. The phenotypic data on patients with CNVs were obtained from the DECIPHER consortium [22]. The DECIPHER website offers information on how researchers can obtain access to this data [68]. It is also possible to visualize individual deletions in the UCSC Genome Browser [69]. For example, the deletion chr19:30682288 to 36367331 (which is the second entry in Additional file 1: Table S2) can be visualized by selecting the human genome assembly of February 2009 (GRCh37/hg19) in the UCSC Browser, entering the search term ‘chr19: 30682288-36367331’, and then setting the DECIPHER track in the section Phenotype and Literature to full, and clicking the refresh button. The individual in question has the id DECIPHER:3776, and by letting the mouse hover over the red bar next to the number 3776 in the browser, the corresponding phenotype terms will be shown. It is not currently possible to download all DECIPHER data from the UCSC Genome Browser.

We used the latest version of the DECIPHER data from 5 April 2013 with 7,535 patients. The patient IDs are represented by increasing numbers and the last patient we analyzed has the ID 273601. The HPO and Uberpheno data are publicly available from the HPO download page [70]. For the analysis described here, we used OBO files and annotation tables from HPO build #856 (9 December 2013) and build #132 (9 December 2013) of the cross-species ontology Uberpheno.

## Additional file

Additional file 1 **Online supplementary material.** **Figure S1.** DNase I hypersensitive sites. **Figure S2.** Overview of TDBD filtering steps. **Figure S3.** TDBD deletions according to number of disrupted domain boundaries. **Figure S4.** Contribution of tissue-specific enhancers to TDBD effect and phenogram score. **Figure S5.** Phenotype explanations using model organism data. **Table S1.** Reproducibility of tissue-specific enhancers. **Table S2.** Summary of TDBD analysis.

## Abbreviations

bp: base pair
CNV: copy-number variation/variant
CTS-DHS: cell-type-specific DHS
DHS: DNase I hypersensitive site
DNase-seq: DNase-sequencing
GDE: gene-dosage effect
HPO: Human Phenotype Ontology
kb: kilobase
Mb: megabase
NIH REMC: National Institutes of Health’s Roadmap Epigenomics Mapping Consortium
TDB: topological domain boundary
TDBD: TDB disruption
WS: Williams syndrome
WTCCC2: Wellcome Trust Case Control Consortium 2
