# Supplementary material for: Deletions of chromosomal regulatory boundaries are associated with congenital disease
Source: Genome Biol. 2014 Sep 4;15(9):423. doi: 10.1186/s13059-014-0423-1 (PMC4180961; doi:10.1186/s13059-014-0423-1)
Supplement: Supplementary file 1 — Online supplementary material. Figure S1. DNase I hypersensitive sites. Figure S2. Overview of TDBD filtering steps. Figure S3. TDBD deletions according to number of disrupted domain boundaries. Figure S4. Contribution of tissue-specific enhancers to TDBD effect and phenogram score. Figure S5. Phenotype explanations using model organism data. Table S1. Reproducibility of tissue-specific enhancers. Table S2. Summary of TDBD analysis. [file 13059_2014_423_MOESM1_ESM.pdf]

Deletions of Chromosomal Regulatory Boundaries are Associated with Congenital Disease: Online Supplementary Material

Ibn-Salem J et al.

Contents

Fig. S1. DNase I hypersensitive sites . . . 2
Fig. S2. Overview of TDBD filtering steps . . . 3
Fig. S3. TDBD deletions according to number of disrupted domain boundaries . . . 4
Fig. S4. Contribution of tissue specific enhancers to *TDBD* effect and phenogram score . . . 5
Fig. S5. Phenotype explanations using model organism data . . . 6
Table. S1. Reproducibility of tissue-specific enhancers . . . 7
Table S2. Summary of TDBD Analysis . . . 8

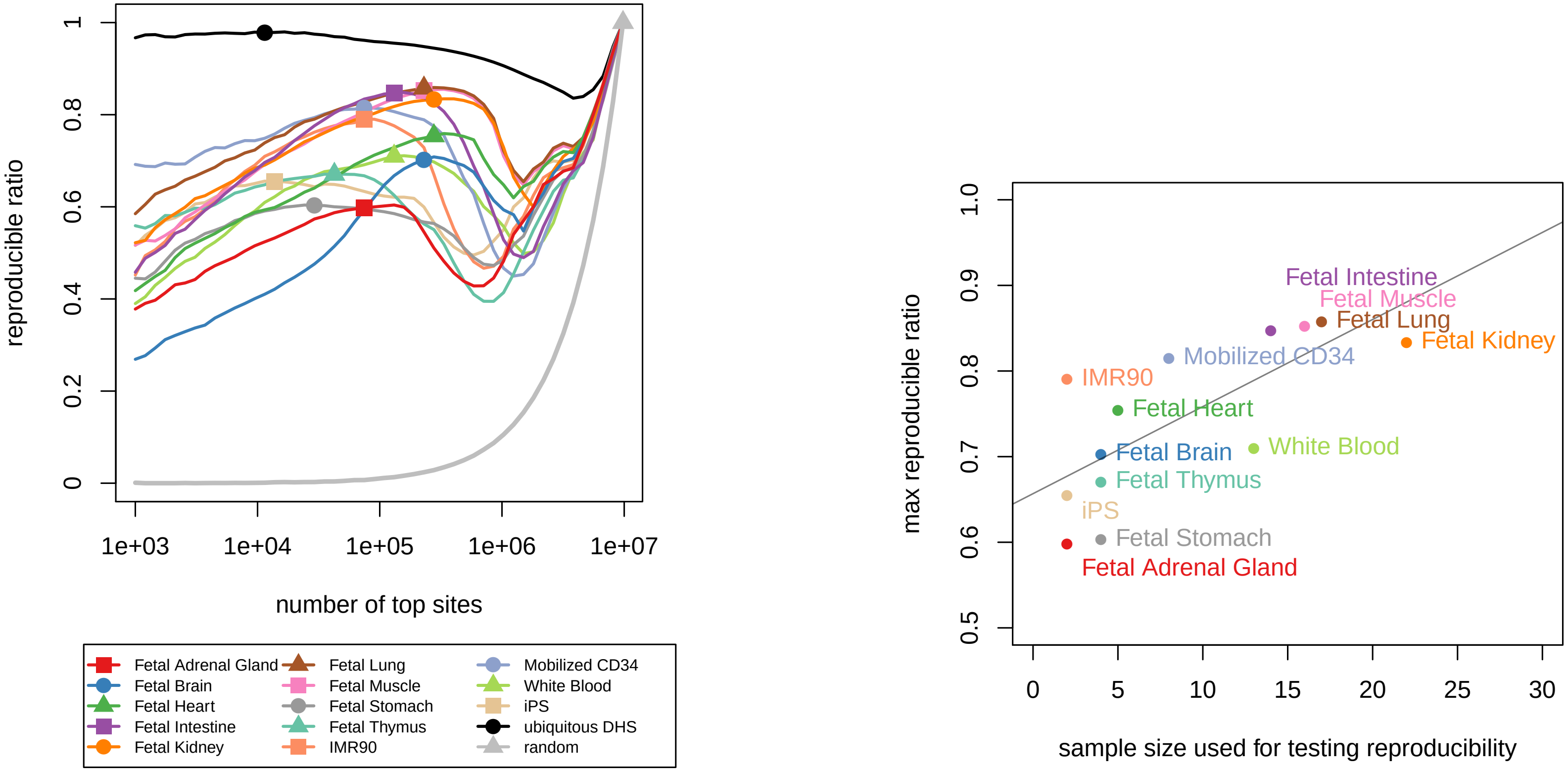

Figure S1. DNase I hypersensitive sites. In order to determine the extent of cell-type-specific DHS (CTS-DHS) per cell type reflected in the data, we examined the reproducibility of our rankings. We split samples from all tissues into two groups, and derived rankings of tissue specificity as described above for each group separately. For the top n windows from the two groups, we calculated the proportion of windows which are shared. For increasing n, we observed maxima of the reproducible ratio for each tissue and define this n as the number of most reproducible CTS-DHS. The cutoff defined by reproducibility depends on the number of samples per tissue, with more samples leading to higher proportions of shared rankings (Supplementary Fig. S1B). The reproducibility cutoff is, except in the case of iPS cells, more conservative than using a 0.05 false-discovery rate (FDR) cutoff of Benjamini-Hochberg corrected t-distribution p-values (Supplementary Table S1). The overlap of top ranked windows is highly significant when compared to the number expected between random rankings ($p < 2.2 \times 10^{-16}$ using Fisher's exact test). Note that only the ten tissues most clearly corresponding to major phenotypic categories were used for the remaining analysis (see Table 2 of main manuscript). **(A)** Ranking of cell-type-specificity by reproducibility. All samples are split into two equally-stratified groups, and for a given number n of top sites (x-axis), the ratio of reproducible top-ranked sites is computed (y-axis) A maxima of reproducible rankings occurs for most cell types at around 100,000 sites. **(B)** Reproducibility and sample size. The maxima of reproducibility (shown in Panel A of this Figure) depends on the sample size used for generating rankings (x-axis). Here the sample size indicated is one half of the total samples, as the data was split for measuring reproducibility.

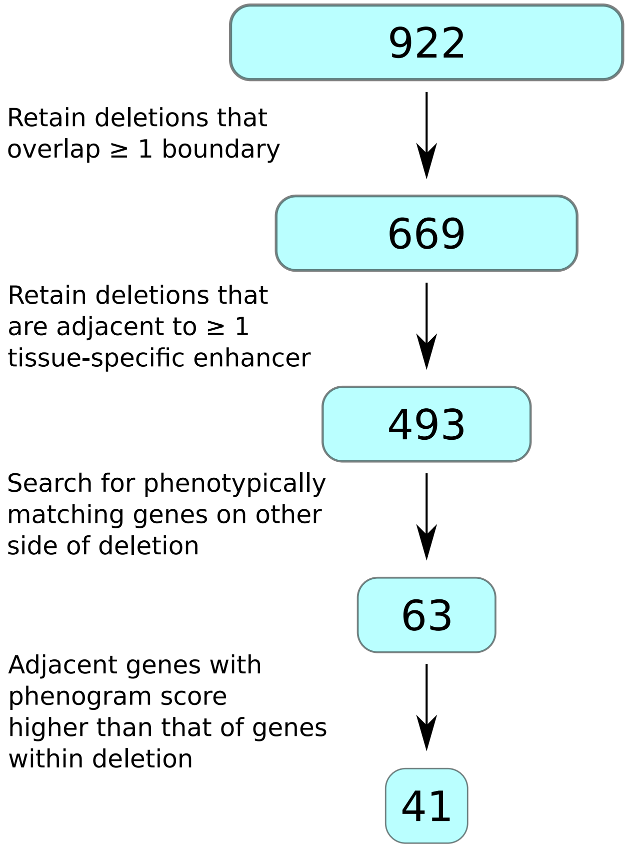

Figure S2. The various steps of the analysis are symbolized. 2300 deletions were obtained from DECIPHER. 922 of them could be assigned to a unique target phenotype term, and 699 were also adjacent to a tissue-specific enhancer. 63 of these cases showed computational evidence of topological domain boundary disruption on the basis of phenotypic similarity between the deletion and a gene on one side of the deletion and the presence of an enhancer specific for the affected tissue type on the other side of the deletion. In 41 of these cases, the phenotypic similarity was higher for the TDBD than for a potential gene-dosage effect. These CNVs are listed in table **S2**.

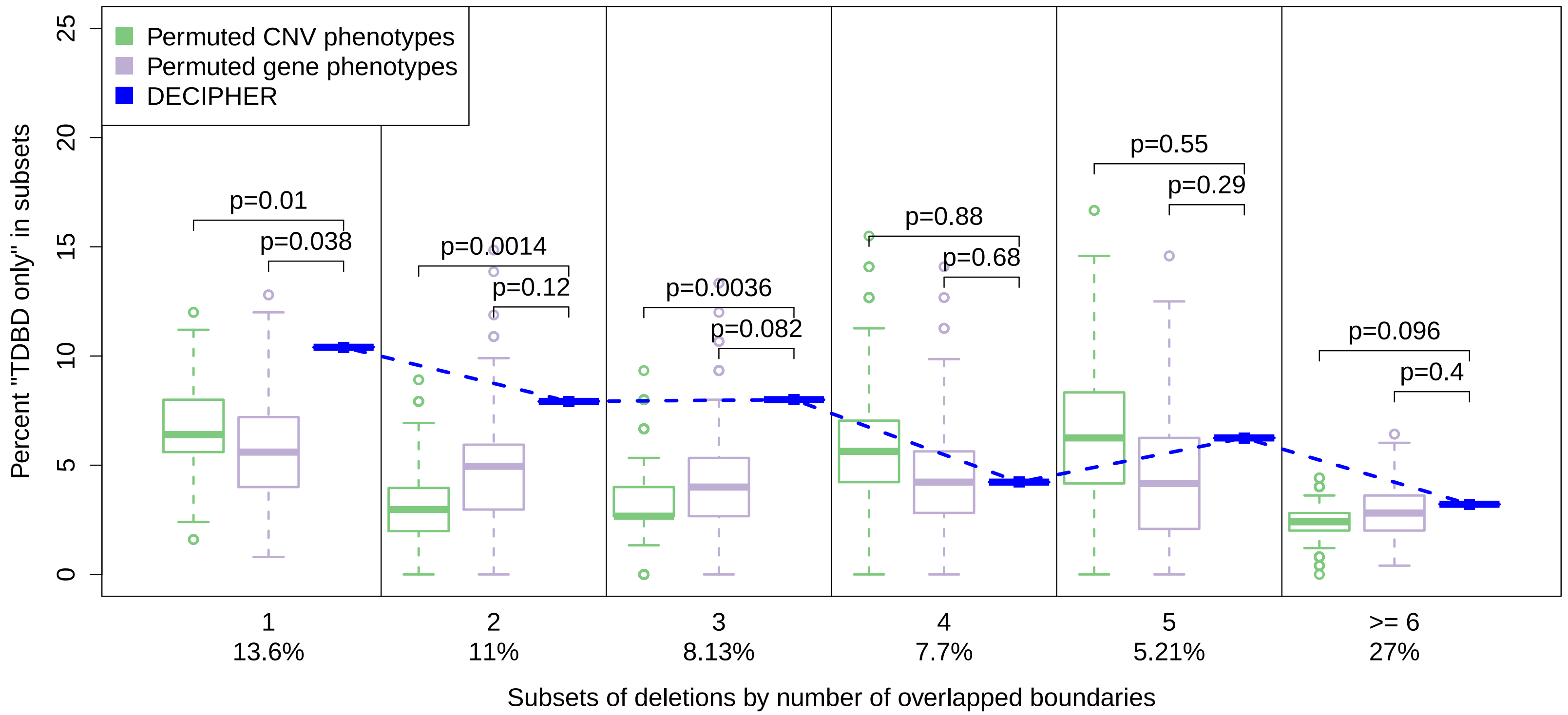

Figure S3. Percentage of cases with only *TDBD* pathogenicity separated in subsets according to the number of domain boundaries disrupted by the deletion. The comparison is to the percentage of expected effects by randomly permuted phenotype annotation of patients (green) and permutated phenotypes associated to genes (purple). The p-value is the fraction of randomizations for which the percentage of TDBD hits observed is at least as high as for the DECIPHER deletions. Small DECIPHER deletions, that overlap only view boundary show higher rates of TDBD mechanism than larger deletion with many boundaries.

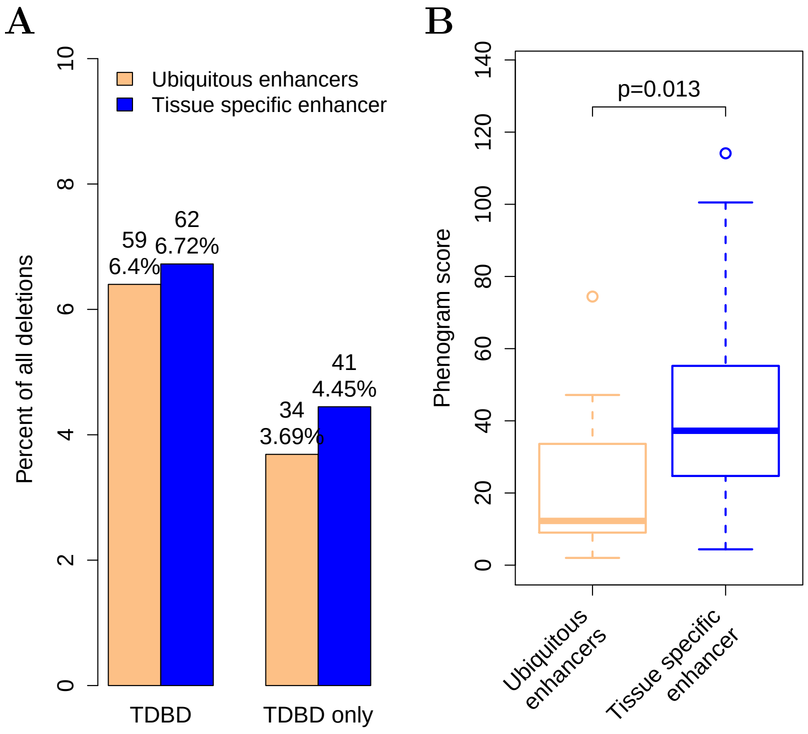

Figure S4. Contribution of tissue specific enhancers to *TDBD* effect and Phenogram score. **(A)** The percent of CNVs with *TDBD* and *TDBD only* effect mechanism by using the ubiquitous DNase hyper sensitive sites (DHS) (orange) or tissue specific DHS (blue) as enhancers for the *TDBD* model. **(B)** The similarity between phenotypes of the patients and genes adjacent to the deletions are compared as phenogram score for *TDBD only* events with ubiquitous and tissue specific enhancers. *TDBD* deletions with tissue specific enhancers have significantly higher phenogram scores (Wilcoxon p-value $= 0.013$).

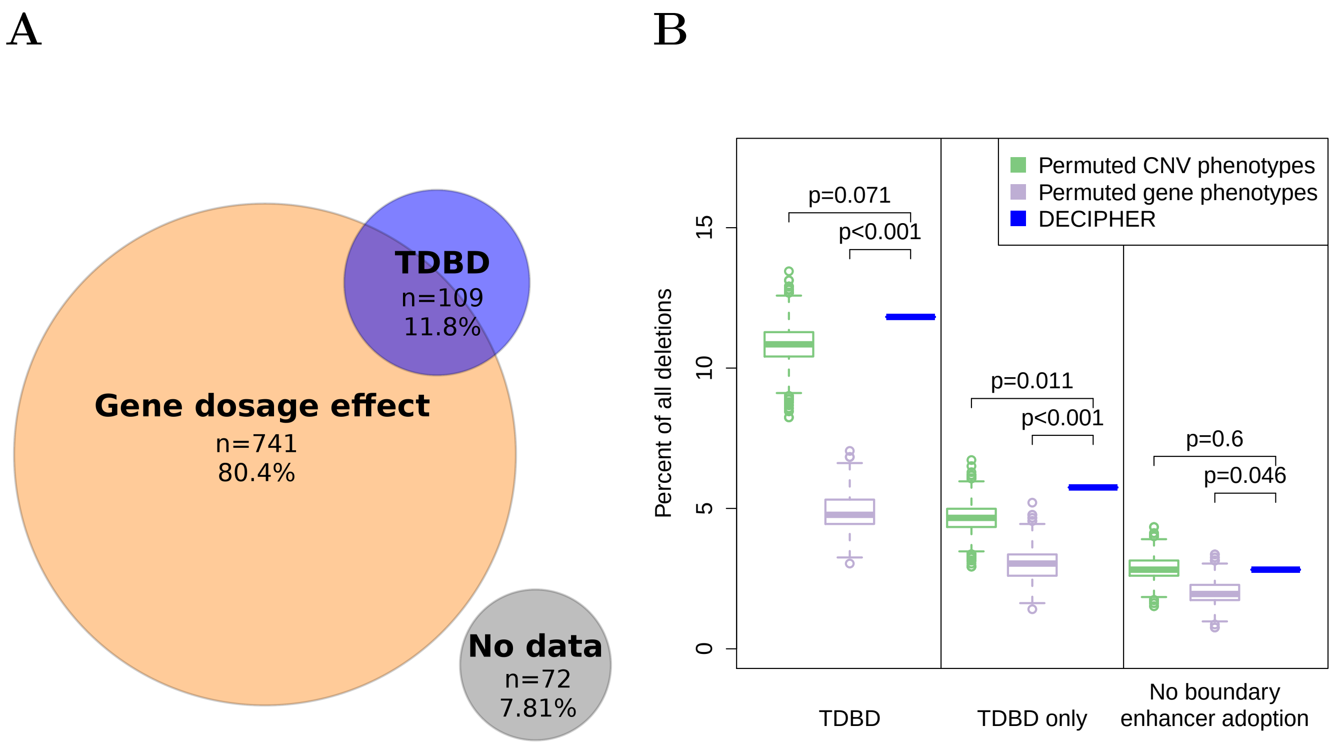

Figure S5. **(A)** Phenotype explanation of 922 CNVs as gene dosage effect (GDE) or topological domain boundary disruption (TDBD). The analysis is analogous to the analysis shown in Fig. 4 in the main text except that the cross-species ontology uberpheno [1] is used for the phenotypic analysis. **(B)** The fraction of CNVs assigned to the indicated categories for the DECIPHER data and the phenotype-shuffled data.

cell	reproducibility maxima cutoff	reproducibility maxima merged	Benjamini-Hochberg FDR < 0.05 cutoff	Benjamini-Hochberg FDR < 0.05 merged
Fetal Adrenal Gland	85417	53382	535185	347858
Fetal Brain	272601	130381	689249	293774
Fetal Heart	283063	108812	540534	187709
Fetal Intestine	139108	65296	537340	249035
Fetal Kidney	288783	126542	704496	262708
Fetal Lung	258375	107664	664150	238944
Fetal Muscle	272601	107115	662978	229239
Fetal Stomach	30434	19189	285236	166037
Fetal Thymus	43405	23650	506287	303871
IMR90	77720	52594	565900	402715
Mobilized CD34	88496	52388	636117	378429
White Blood	155319	81993	1123209	593020
iPS	15229	11146	0	0

Table S1. Cutoffs from reproducibility curves and Benjamini-Hochberg correction of t-tests, before and after merging adjacent windows

Table S2. Summary of TDBD analysis

Nr.	Deletion	CNV Phenotype	GDE	TDBD	Enhancer	Gene Phenotype
1	chr3:181692255–185969168<br>(DECIPHER:1495)	Cryptorchidism<br>**Microcephaly**<br>Delayed eruption of teeth<br>Abnormality of dental morphology<br>Stenosis of the external auditory canal<br>Feeding difficulties in infancy<br>Micropenis<br>**Intellectual disability**<br>Small for gestational age	28.1<br>(58 genes with 165 coding and 29 noncoding transcripts)	42.0 (n=7)	brain<br>n=20 (5')<br>n=7 (3')	*SOX2* (5')<br>Microcephaly<br>Aplasia/Hypoplasia of the corpus callosum<br>Agenesis of corpus callosum<br>Hypothalamic hamartoma,HP:0002079<br>Hypoplasia of the corpus callosum
1	*SOX2* (Gene ID:6657): Optic nerve hypoplasia and abnormalities of the central nervous system [MIM:206900]					
2	chr19:30682288-36367331<br>(DECIPHER:3776)	**Microcephaly**<br>Short stature<br>Feeding difficulties in infancy<br>Aplasia cutis congenita of scalp<br>**Intellectual disability**<br>Hypospadias	33.8<br>(135 genes with 238 coding and 70 noncoding transcripts)	40.4 (n=8)	brain<br>n=8 (5')	*WDR62* (3')<br>Hypoplasia of the corpus callosum<br>Seizures<br>Intellectual disability<br>Global developmental delay<br>Schizencephaly<br>Microcephaly<br>Polymicrogyria<br>Lissencephaly<br>Pachygyria<br>*SDHAF1* (3')<br>Progressive Leukoencephalopathy<br>Developmental regression<br>Seizures<br>*TYROBP* (3')<br>Cerebral cortical atrophy<br>Caudate atrophy<br>Developmental regression<br>Cerebral calcification
2	*WDR62* (Gene ID:284403): Microcephaly 2, primary, autosomal recessive, with or without cortical malformations [MIM:604317]					
2	*SDHAF1* (Gene ID:644096): Mitochondrial complex II deficiency [MIM:252011]					
2	*TYROBP* (Gene ID:7305): Nasu-Hakola disease [MIM:221770]					
3	chr15:75600115-76019989<br>DECIPHER:255342	**Microcephaly**<br>**Intellectual disability**	0<br>(12 genes with 28 coding and 4 noncoding transcripts)	9.6 (n=2)	brain<br>n=3 (5')<br>n=1 (3')	*ETFA* (3')<br>Pachygyria
3	*ETFA* (Gene ID:2108): Glutaric acidemia IIA [MIM:231680]					

Continued on next page

Table S2 – *Continued from previous page*

Nr.	Deletion	CNV Phenotype	GDE	TDBD	Enhancer	Gene Phenotype
4	chr6:79324861-88043414<br>DECIPHER:1878	Abnormality of the kidney<br>2-3 toe syndactyly<br>Obesity<br>**Hypoplasia of the corpus callosum**<br>Macrocephaly<br>Umbilical hernia<br>Cataract<br>Epicanthus<br>Macrotia<br>Downslanted palpebral fissures<br>High palate<br>Short palm<br>Blepharophimosis<br>Hearing impairment<br>**Behavioural/Psychiatric Abnormality**<br>**Intellectual disability**<br>Short foot<br>Abnormality of the forehead	16.0<br>(42 genes with 95 coding transcripts and 15 noncoding transcripts)	25.9 (n=8)	brain<br>n=13 (5')<br>n=35 (3')	*RARS2* (3')<br>Cerebral cortical atrophy<br>Progressive microcephaly
4	*RARS2* (Gene ID:57038): Pontocerebellar hypoplasia, type 6 [MIM:611523]					
5	chr6:152052838-157013183<br>DECIPHER:1911	**Intellectual disability**<br>**Hypoplasia of the corpus callosum**	0.6<br>(20 genes with 95 coding and 17 noncoding transcripts)	9.9 (n=5)	brain<br>n=2 (5')<br>n=4 (3')	*RMND1* (5')<br>Cerebral cortical atrophy<br>Pachygyria<br>Hypoplasia of the corpus callosum<br>*ARID1B* (3')<br>Microcephaly
5	*RMND1* (Gene ID:55005): Combined oxidative phosphorylation deficiency 11 [MIM:614922]					
5	*ARID1B* (Gene ID:57492): Mental retardation, autosomal dominant 12 [MIM:614562]					
6	chr12:23924732-25330906<br>DECIPHER:253839	**Microcephaly**<br>**Intellectual disability**	0<br>(7 genes with 26 coding and 5 noncoding transcripts)	57.7 (n=3)	brain<br>n=20 (5')<br>n=8 (3')	*KRAS* (3')<br>Porencephaly<br>Hemimegalencephaly<br>Global developmental delay
6	*KRAS* (Gene ID:up=NA, down=3845): Cardiofaciocutaneous syndrome 2 [MIM:615278], Noonan syndrome 3 [MIM:609942]					

Continued on next page

Nr.	Deletion	CNV Phenotype	GDE	TDBD	Enhancer	Gene Phenotype
7	chr14:29904720-30316660<br>DECIPHER:252353	**Microcephaly**<br>**Hypoplasia of the corpus callosum**<br>Prominent nasal bridge<br>Blepharophimosis<br>Intrauterine growth retardation<br>Feeding difficulties in infancy<br>Thick upper lip vermilion<br>Upslanted palpebral fissure<br>**Intellectual disability**<br>Ventricular septal defect<br>Short philtrum	0<br>(4 genes with 1 coding and 4 noncoding transcripts)	76.3 (n=1)	brain<br>n= 80 (5')<br>n=40 (3')	*FOXG1* (5')<br>Pachygyria<br>Microcephaly<br>Hypoplasia of the corpus callosum<br>Cerebral cortical atrophy<br>Aplasia/Hypoplasia of the corpus callosum<br>Progressive microcephaly
7	*FOXG1* (Gene ID:2290): Rett syndrome, congenital variant [MIM:613454]					
8	chr3:179971262-180988013<br>DECIPHER:249752	**Microcephaly**<br>Talipes equinovarus	1.8<br>(8 genes with 14 coding and 10 noncoding transcripts)	24.0 (n=1)	brain<br>n=11 (5')<br>n=19 (3')	*SOX2* (3')<br>Microcephaly<br>Aplasia/Hypoplasia of the corpus callosum<br>Agenesis of corpus callosum<br>Hypothalamic hamartoma<br>Hypoplasia of the corpus callosum
8	*SOX2* (Gene ID:6657): Optic nerve hypoplasia and abnormalities of the central nervous system [MIM:206900]					
9	chr15:49896865-60460116<br>DECIPHER:965	**Intellectual disability**<br>**Hypoplasia of the corpus callosum**	11.5<br>(178 genes with 210 coding and 148 noncoding transcripts)	42.3<br>(n=13)	brain<br>n= 2 (3')	*CEP152* (5')<br>Abnormal cortical gyration<br>Microcephaly
9	*CEP152*: autosomal recessive primary microcephaly 9 [MIM:614852] and Seckel syndrome 5 [MIM:613823]					
10	chr5:12284356-26988484<br>DECIPHER:250008	Micropenis<br>**Microcephaly**<br>Obesity	23.4<br>(35 genes with 40 coding and 34 noncoding transcripts)	46.3<br>(n=12)	brain<br>n=62 (5')<br>n=6 (3')	*CTNND2* (5')<br>Microcephaly
10	*CTNND2* (Gene ID:1501): Mental retardation in cri-du-chat syndrome [MIM:123450]					
11	chr9:98696759-105723255<br>DECIPHER:253335	Absent nipples<br>**Microcephaly**<br>2-3 toe syndactyly<br>Dysarthria<br>Diabetes mellitus<br>Deeply set eye<br>**Intellectual disability**<br>Abnormality of the philtrum	30,5453<br>(85 genes with 134 coding and 68 noncoding transcripts)	100,0736<br>(n=9)	brain<br>n=4 (3')	*PTCH1* (5')<br>Semilobar holoprosencephaly<br>Aplasia/Hypoplasia of the corpus callosum<br>Cerebral calcification<br>Calcification of falx cerebri<br>Agenesis of corpus callosum<br>Abnormality of the thalamus
11	*PTCH1* (Gene ID: 5727): Holoprosencephaly-7 [MIM:610828]					

Continued on next page

Nr.	Deletion	CNV Phenotype	GDE	TDBD	Enhancer	Gene Phenotype
12	chr14:29695258-30872374<br>DECIPHER:3810	**Microcephaly**<br>**Hypoplasia of the corpus callosum**<br>Bruxism<br>**Delayed speech and language development**<br>Medial flaring of the eyebrow<br>Overlapping toe<br>Short stature<br>Flexion contracture<br>Short nose<br>**Seizures**<br>Feeding difficulties in infancy<br>Short toe<br>**Intellectual disability**	0<br>(4 genes with 1 coding and 4 noncoding transcripts)	85.7 (n=1)	brain<br>n=42 (5')<br>n=7 (3')	*FOXG1* (5')<br>Pachygyria<br>Microcephaly<br>Hypoplasia of the corpus callosum<br>Cerebral cortical atrophy<br>Aplasia/Hypoplasia of the corpus callosum<br>Progressive microcephaly
12	*FOXG1* (Gene ID: 2290): Rett syndrome, congenital variant [MIM:613454]					
13	chrX:6495281-8221971<br>DECIPHER:1585	Abnormality of the palmar creases<br>**Hypoplasia of the corpus callosum**<br>**Intellectual disability**<br>Posteriorly rotated ears<br>Pointed chin<br>High palate<br>Hypertelorism<br>Abnormality of the hair<br>Deep plantar creases<br>Long face	9.9<br>(7 genes with 11 coding and 5 noncoding transcripts)	13.0 (n=2)	brain<br>n=9 (5')<br>n=3 (3')	*KAL1* (3')<br>Hypothalamic gonadotropin-releasing hormone (GNRH) deficiency<br>Ataxia
13	*KAL1* (Gene ID: 3730): Hypogonadotropic hypogonadism 1 with or without anosmia (Kallmann syndrome 1) [MIM:308700]					
14	chr14:29695258-30872374<br>DECIPHER:248405	**Microcephaly**<br>**Intellectual disability**	0<br>(4 genes with 1 coding and 4 noncoding transcripts)	47,7919 (n=1)	brain<br>n=42 (5')<br>n=7 (3')	*FOXG1* (5')<br>Pachygyria<br>Microcephaly<br>Hypoplasia of the corpus callosum<br>Cerebral cortical atrophy<br>Aplasia/Hypoplasia of the corpus callosum<br>Progressive microcephaly
14	*FOXG1* (Gene ID:2290): Rett syndrome, congenital variant [MIM:613454]					
15	chr21:27991572-36610975<br>DECIPHER:249587	**Microcephaly**<br>**Hypoplasia of the corpus callosum**<br>**Cerebral atrophy**<br>Slender build<br>Pulmonic stenosis<br>Muscular hypotonia<br>**Intellectual disability**<br>Ventricular septal defect	12.4<br>(115 genes with 198 coding and 68 noncoding transcripts)	20.2 (n=12)	brain<br>n=9 (5')<br>n=3 (3')	*APP* (5')<br>Cerebral calcification<br>Neurofibrillary tangles<br>Dementia<br>Parkinsonism

Continued on next page

Table S2 – *Continued from previous page*

Nr.	Deletion	CNV Phenotype	GDE	TDBD	Enhancer	Gene Phenotype
15	*APP* (Entrez ID:351):					
16	chr9:108940763-110561397<br>DECIPHER:261011	Ptosis<br>Muscular hypotonia<br>**Hypoplasia of the corpus callosum**	0<br>(9 genes with 14 coding and 7 noncoding transcripts)	54.8 (n=1)	brain<br>n=7 (5')<br>n=10 (3')	*FKTN* (5')<br>Type II lissencephaly<br>Pachygyria<br>Aplasia/Hypoplasia of the corpus callosum<br>Agenesis of corpus callosum<br>Polymicrogyria
16	*FKTN* (Gene ID:2218): Muscular dystrophy-dystroglycanopathy (congenital with brain and eye anomalies), type A, 4 [MIM:607440]					
17	chr16:14622055-17409257<br>DECIPHER:249627	Median cleft lip<br>Abnormality of the pinna<br>Cleft palate<br>Stenosis of the external auditory canal<br>Preauricular skin tag<br>**Holoprosencephaly**<br>Aplasia of the nose<br>Cyclopia	25.2591<br>(35 genes with 73 coding and 24 noncoding transcripts)	33.7 (n=4)	brain<br>n=23 (3')	*ERCC4* (5')<br>Microcephaly<br>Intellectual disability<br>Brain atrophy
17	*ERCC4* (Gene ID:2072): Fanconi anemia, complementation group Q [MIM:133520], Xeroderma pigmentosum, type F/Cockayne syndrome [MIM:133520]					
18	chr16:14622055-17409257<br>DECIPHER:249447	**Spasticity**<br>**Hypoplasia of the corpus callosum**<br>**Dystonia**	8.0<br>(35 genes with 73 coding and 24 noncoding transcripts)	10.2 (n=4)	brain<br>n=23 (3')	*ERCC4* (5')<br>Microcephaly<br>Intellectual disability<br>Brain atrophy
18	*ERCC4* (Gene ID:2072): Fanconi anemia, complementation group Q [MIM:133520], Xeroderma pigmentosum, type F/Cockayne syndrome [MIM:133520]					
19	chr5:130931-36780974<br>DECIPHER:256304	**Atrioventricular canal defect**	4.6<br>(166 genes with 274 coding and 139 noncoding transcripts)	4.4223<br>(n=32)	heart<br>n=5 (5')	*NIPBL* (3')<br>Ventricular septal defect
19	*NIPBL* (Gene ID:25836): Cornelia de Lange syndrome 1 [MIM:122470]					

Continued on next page

Table S2 – *Continued from previous page*

Nr.	Deletion	CNV Phenotype	GDE	TDBD	Enhancer	Gene Phenotype
20	chr19:16517519-17477318<br>DECIPHER:4101	Preaxial hand polydactyly<br>Hypertelorism<br>Microcephaly<br>Strabismus<br>Split hand<br>Delayed speech and language development<br>Bifid nail<br>Micropenis<br>Wide mouth<br>Cutaneous finger syndactyly<br>Split foot<br>Hypopigmentation of the skin<br>Broad face<br>**Tetralogy of Fallot**<br>Deeply set eye<br>Anteverted nares<br>Upslanted palpebral fissure<br>Intellectual disability<br>Brachycephaly<br>Abnormality of the philtrum<br>Widely spaced teeth	1.8<br>(27 genes with 65 coding and 11 noncoding transcripts)	56.2115<br>(n=2)	heart<br>n=6 (5')	*PIK3R2* (3')<br>Mitral regurgitation<br>Abnormality of the mitral valve<br>Ventricular septal defect<br>Defect in the atrial septum
20	*PIK3R2* (Gene ID:5296): Megalencephaly-polymicrogyria-polydactyly-hydrocephalus syndrome [MIM:603387]					
21	chr3:8330426-9910334<br>DECIPHER:253231	Downturned corners of mouth<br>Seizures<br>Delayed speech and language development<br>Ptosis<br>Overlapping toe<br>Muscular hypotonia<br>Hypertelorism<br>Feeding difficulties in infancy<br>**Atrioventricular canal defect**<br>Intellectual disability	24.2<br>(30 genes with 85 coding and 15 noncoding transcripts)	37.3 (n=3)	heart<br>n=4 (5')	*CRELD1* (3')<br>Dextrocardia<br>Pulmonary artery atresia<br>Right aortic arch with mirror image branching<br>*FANCD2* (3')<br>Tetralogy of Fallot<br>Abnormality of the aorta<br>Defect in the atrial septum
21	*CRELD1* Atrioventricular septal defect, partial, with heterotaxy syndrome [MIM:606217]					
21	*FANCD2* (Gene ID:2177): Fanconi anemia, complementation group D2 [MIM:227646]					
22	chr22:21105634-22423216<br>DECIPHER:254238	Patent ductus arteriosus<br>Microcephaly<br>Prenatal short stature<br>**Ventricular septal defect**	21.5<br>(54 genes with 63 coding and 43 noncoding transcripts)	35.9 (n=2)	heart<br>n=3 (5')<br>n=5 (3')	*SMARCB1* (3')<br>Malformation of the heart and great vessels
22	*SMARCB1* (Gene ID:6598): Coffin Siris syndrome [Tsurusaki Y et al. (2012), *Nat Genet* **44**:376-8.]					

Continued on next page

Table S2 – *Continued from previous page*

Nr.	Deletion	CNV Phenotype	GDE	TDBD	Enhancer	Gene Phenotype
23	chr19:15978604-17500427<br>DECIPHER:262142	Clinodactyly of the 5th finger<br>Proportionate short stature<br>Low anterior hairline<br>Brachydactyly syndrome<br>Bifid uvula<br>Epicanthus<br>Short nose<br>Long philtrum<br>Intellectual disability<br>**Ventricular septal defect**	1.8<br>(74 genes with 89 coding and 56 noncoding transcripts)	42.6 (n=2)	heart<br>n=4 (5')	*PIK3R2* (3')<br>Mitral regurgitation<br>Abnormality of the mitral valve<br>Ventricular septal defect<br>Defect in the atrial septum
23	*PIK3R2* (Gene ID:5296): Megalencephaly-polymicrogyria-polydactyly-hydrocephalus syndrome [MIM:603387]					
24	chr22:21075575-22467350<br>DECIPHER:2366	Microcephaly<br>Proportionate short stature<br>Prenatal short stature<br>Brachycephaly<br>**Defect in the atrial septum**<br>**Ventricular septal defect**<br>Anal atresia	23.5<br>(55 genes with 64 coding and 48 noncoding transcripts)	37.5 (n=2)	heart<br>n=2 (5')<br>n=5 (3')	*SMARCB1* (3')<br>Malformation of the heart and great vessels
24	*SMARCB1* (Gene ID:6598): Coffin Siris syndrome [Tsurusaki Y et al. (2012), *Nat Genet* **44**:376-8.]					
25	chr10:118907361-122761687<br>DECIPHER:262197	**Renal agenesis**<br>Intellectual disability<br>Abnormality of the genital system<br>Scoliosis	9.0<br>(34 genes with 56 coding and 23 noncoding transcripts)	114.6<br>(n=5)	kidney<br>n=9 (5')<br>n=30 (3')	*FGFR2* (3')<br>Nephrosclerosis<br>Renal agenesis<br>Kidney malformation<br>Hydronephrosis
25	*FGFR2* (Gene ID:2263): LADD syndrome [MIM:149730], Antley-Bixler syndrome without genital anomalies or disordered steroidogenesis [MIM:207410]					
26	chr20:48461499-50342355<br>DECIPHER:257542	Cryptorchidism<br>Short nose<br>Talipes equinovarus<br>Sclerocornea<br>Long philtrum<br>Anteverted nares<br>**Multiple renal cysts**<br>Intellectual disability<br>Frontal bossing	21,828<br>(24 genes with 57 coding and 11 noncoding transcripts)	93,5562<br>(n=4)	kidney<br>n=1 (5')<br>n=6 (3')	*SALL4* (3')<br>Abnormal localization of kidneys<br>Renal agenesis<br>Renal malrotation<br>Crossed fused renal ectopia<br>Horseshoe kidney<br>Hydronephrosis<br>Renal hypoplasia<br>Renal hypoplasia/aplasia
26	*SALL4* (Gene ID: 57167): Duane-radial ray syndrome [MIM:607323]					

Continued on next page

Table S2 – *Continued from previous page*

Nr.	Deletion	CNV Phenotype	GDE	TDBD	Enhancer	Gene Phenotype
27	chrX:101688057-103770041<br>DECIPHER:249664	Joint laxity<br>Soft skin<br>Brachycephaly<br>Skin dimples<br>Constipation<br>**Muscular hypotonia**<br>Strabismus<br>Prominent fingertip pads<br>Intellectual disability<br>Depressed nasal bridge	33.4<br>(42 genes with 118 coding and 16 noncoding transcripts)	35.2 (n=3)	muscle<br>n=45 (3')	*GLA* (5')<br>Fasciculations<br>Myalgia<br>Muscle cramps
27	*GLA* (Gene ID: 2717): Fabry disease [MIM:301500]					
28	chr2:72287394-73070977<br>DECIPHER:249203	**Facial palsy**<br>Autism<br>Intellectual disability<br>Prominent ears	2,2825<br>(4 genes with 5 coding and 2 noncoding transcripts)	15,8853<br>(n=1)	muscle<br>n=10 (5')	*SPR* (3')<br>Choreoathetosis<br>Muscular hypotonia of the trunk
28	*SPR* (Gene ID:6697):Dystonia, dopa-responsive, due to sepiapterin reductase deficiency 612716					
29	chr10:130955710-135397841<br>DECIPHER:249776	Vesicoureteral reflux<br>Delayed speech and language development<br>Skin dimples<br>Fine hair<br>Spasticity<br>**Muscular hypotonia**<br>Strabismus<br>Deeply set eye<br>Micrognathia	0<br>(47 genes with 86 coding and 28 noncoding transcripts)	7.5 (n=2)	muscle<br>n=107 (5')	*DUX4* (3')<br>Amyotrophy<br>EMG abnormality
29	*DUX4* (Gene ID:22947): *increased expression in* Facioscapulohumeral muscular dystrophy 1 [MIM:158900]					

Continued on next page

Table S2 – *Continued from previous page*

Nr.	Deletion	CNV Phenotype	GDE	TDBD	Enhancer	Gene Phenotype
30	chr2:170762840-175523183<br>DECIPHER:250211	Abnormality of dental enamel<br>Abnormality of the palmar creases<br>Clinodactyly of the 5th finger<br>2-3 toe syndactyly<br>Thickened ears<br>Tapered finger<br>Short neck<br>Posteriorly rotated ears<br>Nail dysplasia<br>Microcephaly<br>Downslanted palpebral fissures<br>**Muscular hypotonia**<br>High palate<br>Obesity<br>Feeding difficulties in infancy<br>Proportionate short stature<br>Facial asymmetry<br>Short toe<br>Intellectual disability	31.1<br>(39 genes with 115 coding and 21 noncoding transcripts)	75.9 (n=5)	muscle<br>n=2 (5')<br>n=2 (3')	*CHRNA1* (3')<br>Gower sign<br>Intermittent episodes of respiratory insufficiency due to muscle weakness<br>Bulbar palsy<br>Camptodactyly of finger<br>Easy fatigability<br>Respiratory insufficiency due to muscle weakness<br>Fatigable weakness<br>Flexion contracture<br>Muscular hypotonia<br>Arthrogryposis multiplex congenita<br>Generalized muscle weakness<br>Generalized amyoplasia<br>Type 2 muscle fiber atrophy
30	*CHRNA1* (Gene ID:1134): Multiple pterygium syndrome, lethal type [MIM:253290], Myasthenic syndrome, fast-channel congenital [MIM:608930] Myasthenic syndrome, slow-channel congenital [601462]					
31	chr14:29538005-30335064<br>DECIPHER:260836	Intellectual disability<br>Microcephaly<br>**Muscular hypotonia**	0<br>(4 genes with 1 coding and 4 noncoding transcripts)	52.5 (n=1)	muscle<br>n=4 (5')<br>n=16 (3')	*FOXG1* (5')<br>Camptodactyly of finger<br>Athetosis<br>Muscular hypotonia<br>Chorea<br>Neonatal hypotonia
31	*FOXG1* (Gene ID: 2290): Rett syndrome, congenital variant [MIM:613454]					
32	chr10:10901193-13286601<br>DECIPHER:258006	Delayed speech and language development<br>**Camptodactyly of finger**	3.3<br>(24 genes with 40 coding and 20 noncoding transcripts)	20.5 (n=3)	muscle<br>n=15 (5')<br>n=2 (3')	*PHYH* (3')<br>Limb muscle weakness<br>Amyotrophy<br>Muscular hypotonia<br>Camptodactyly (feet)
32	*PHYH* (Gene ID:5264): Refsum disease [MIM:266500]					
33	chr5:88232587-90181833<br>DECIPHER:251716	**Muscular hypotonia**<br>Intellectual disability<br>Seizures	6.4<br>(7 genes with 10 coding and 4 noncoding transcripts)	12.2 (n=1)	muscle<br>n=3 (3')	*MEF2C* (5')<br>Muscular hypotonia

Continued on next page

Table S2 – *Continued from previous page*

Nr.	Deletion	CNV Phenotype	GDE	TDBD	Enhancer	Gene Phenotype
33	*MEF2C* (Gene ID:4208): Mental retardation, stereotypic movements, epilepsy, and/or cerebral malformations [MIM:613443]					
34	chr2:238586881-239426490<br>DECIPHER:2170	Microcephaly<br>Macrotia<br>Deeply set eye<br>Joint laxity<br>Short nose<br>Clinodactyly of the 5th finger<br>**Muscular hypotonia**<br>Feeding difficulties in infancy<br>Stereotypic behavior<br>Narrow forehead<br>Intellectual disability	1.7<br>(18 genes with 40 coding and 7 noncoding transcripts)	65.1 (n=1)	muscle<br>n=3 (5')<br>n=17 (3')	COL6A3 (5')<br>Elbow flexion contracture<br>Congenital muscular torticollis<br>Distal muscle weakness<br>EMG abnormality<br>Respiratory insufficiency due to muscle weakness<br>Limb-girdle muscle weakness<br>Torticollis<br>Ankle contracture<br>Camptodactyly of finger<br>Neonatal hypotonia<br>Flexion contracture<br>Generalized amyotrophy<br>Increased variability in muscle fiber diameter<br>Facial palsy<br>Type 1 muscle fiber predominance<br>Proximal muscle weakness<br>Muscle fiber necrosis<br>Myopathy<br>*NDUFA10* (3')<br>*HDAC4* (3')<br>Abnormality of the musculature<br>Muscular hypotonia
34	COL6A3 (Gene ID:1293): Bethlem myopathy [MIM:158810] Ullrich congenital muscular dystrophy [MIM:254090]					
34	*NDUFA10* (Gene ID:4705): Leigh syndrome [MIM:256000]					
34	*HDAC4* (Gene ID:9759): Brachydactyly-mental retardation syndrome [MIM:600430]					

Continued on next page

Nr.	Deletion	CNV Phenotype	GDE	TDBD	Enhancer	Gene Phenotype
35	chr17:46485603-48258140<br>DECIPHER:256421	Microcephaly<br>Wide intermamillary distance<br>Curly eyelashes<br>Ptosis<br>Brachycephaly<br>Brachydactyly syndrome<br>Cavernous hemangioma<br>**Flexion contracture**<br>Long eyelashes<br>Pectus excavatum<br>Deep philtrum<br>Sloping forehead<br>Pili torti<br>Small for gestational age<br>Low-set ears	31.1<br>(53 genes with 108 coding and 18 noncoding transcripts)	131.7<br>(n=1)	muscle<br>n=4 (3')	*PNPO* (5')<br>Myoclonus<br>Muscular hypotonia of the trunk
35	*PNPO* (Gene ID:55163): Pyridoxamine 5'-phosphate oxidase deficiency [MIM:610090]					
36	chr10:129690073-135422505<br>DECIPHER:4069	**Elbow flexion contracture**<br>Obesity<br>Widely spaced toes<br>Tapered finger<br>Constipation<br>Downslanted palpebral fissures<br>**Muscular hypotonia**<br>Seizures<br>Narrow forehead<br>Hallux valgus<br>Intellectual disability<br>Long face<br>Cutis marmorata<br>Hypertelorism	0<br>(51 genes with 96 coding and 31 noncoding transcripts)	8,2923<br>(n=3)	muscle<br>n=33 (5')	*DUX4* (3')<br>Amyotrophy<br>EMG abnormality
36	*DUX4* (Gene ID:22947): *increased expression in* Facioscapulohumeral muscular dystrophy 1 [MIM:158900]					
37	chr14:29781404-30552936<br>DECIPHER:258584	Hypertension<br>Hypothyroidism<br>Seizures<br>Delayed speech and language development<br>**Muscular hypotonia**<br>Obesity<br>Behavioural/Psychiatric Abnormality<br>Intellectual disability	0<br>(4 genes with 1 coding and 4 noncoding transcripts)	30,726<br>(n=1)	muscle<br>n=4 (5')<br>n=15 (3')	*FOXG1* (5')<br>Camptodactyly of finger<br>Athetosis<br>Muscular hypotonia<br>Chorea<br>Neonatal hypotonia

Continued on next page

Table S2 – *Continued from previous page*

**Nr.**	**Deletion**	**CNV Phenotype**	**GDE**	**TDBD**	**Enhancer**	**Gene Phenotype**
37	*FOXG1* (Gene ID: 2290): Rett syndrome, congenital variant [MIM:613454]					
38	chr1:11249468-11318672<br>DECIPHER:2215	Ptosis<br>Short stature<br>Recurrent infections<br>High palate<br>**Facial palsy**<br>Intellectual disability<br>Frontal bossing	0<br>(2 genes with 2 coding and 0 noncoding transcripts)	32.9 (n=1)	muscle<br>n=4 (5')	*MTHFR* (3')<br>*PLOD1*(3')<br>*MFN2*(3')<br>Muscular hypotonia<br>Limb muscle weakness<br>Distal muscle weakness<br>Muscle weakness<br>Distal amyotrophy<br>Foot dorsiflexor weakness<br>Proximal muscle weakness
38	*MTHFR* (Gene ID:4524): Homocystinuria due to MTHFR deficiency [MIM:236250]					
38	*PLOD1* (Gene ID:5351): Ehlers-Danlos syndrome, type VI [MIM:225400]					
38	*MFN2* (Gene ID:9927): Charcot-Marie-Tooth disease, type 2A2 [MIM:609260], Hereditary motor and sensory neuropathy VI [MIM:601152]					
39	chr5:111442710-111977000<br>DECIPHER:4691	Delayed speech and language development<br>Facial palsy<br>Limited shoulder movement<br>Amniotic constriction rings of legs	0<br>(4 genes with 4 coding and 3 noncoding transcripts)	2.90 (n=2)	muscle<br>n=12 (5')	*APC* (3')<br>Myalgia
39	*APC* (Gene ID:324):**Desmoid disease, hereditary**					
40	chr8:117622669-120706534<br>DECIPHER:3785	Multiple exostoses<br>2-3 toe syndactyly<br>**Scapular winging**<br>Abnormality of phalanx of finger<br>Asymmetry of the mouth<br>Thick eyebrow<br>Broad nasal tip<br>Wide nasal bridge<br>Distichiasis<br>Intellectual disability	79.1<br>(20 genes with 26 coding and 8 noncoding transcripts)	101.8<br>(n=3)	muscle<br>n=38 (5')<br>n=4 (3')	*TRPS1* (5')<br>Camptodactyly of finger<br>Scapular winging<br>Muscular hypotonia<br>Infantile muscular hypotonia
40	*TRPS1* (Gene ID:7227): Trichorhinophalangeal syndrome, type I 190350					
41	chr19:31139482-32198771<br>DECIPHER:260316	Abnormality of the musculature<br>Delayed speech and language development<br>Autism	0	18.4<br>(n=1)	muscle<br>n=1 (5')<br>n=3(3')	*C19orf12*<br>Distal amyotrophy<br>Distal muscle weakness
41	*C19orf12* (Gene ID:83636): Neurodegeneration with brain iron accumulation 4 [MIM:614298]					

Supplementary Table S2 . The column "Deletion" shows the chromosomal location (in hg19 coordinated) and the DECIPHER id of the individual in whom the indicated deletion was identified. The column "CNV Phenotype" indicates the phenotypic features (using the Human Phenotype Ontology

term names) found in this individual. The column “GDE” indicates the phenotypic similarity score for genes found to be located within the deletion (Gene Dosage Effect, or GDE) together with the total number of genes within the deleted genomic segment. The column “TDBD” indicates the corresponding phenotypic similarity score for genes adjacent to the deletion and located on the “other” side of the deletion compared to the tissue-specific enhancer (note that in a few cases, both tissue-specific enhancers and phenotypically relevant genes are located on both sides of the deletion). This column also indicates the number of topological domain boundaries removed by the deletion (e.g., n=2 means that two boundaries were removed). The column “Enhancer” indicates the type of tissue-specific enhancer being investigated as well as the total number and the location 5’ or 3’ (in chromosomal coordinates) with respect to the deletion. The column “Gene” indicates the gene or genes identified adjacent to the deletion and with ta positive phenotypic similarity score. Finally, the column “Gene Phenotype” indicates the phenotypic features of the monogenic diseases associated with the genes. Separate rows display the monogenic diseases associated with these genes together with references where more information can be found (in most cases, the Online Mendelian Inheritance in Man, or MIM, number is given) The numbers of genes and transcripts was calculated using the UCSC KnownGenes resources together with a program that used Jannovar [2].

References

[1] Köhler, S., Doelken, S.C., Ruef, B.J., Bauer, S., Washington, N., Westerfield, M., Gkoutos, G., Schofield, P., Smedley, D., Lewis, S.E., Robinson, P.N., Mungall, C.J.: Construction and accessibility of a cross-species phenotype ontology along with gene annotations for biomedical research. F1000Res **2**, 30 (2013)

[2] Jäger, M., Wang, K., Bauer, S., Smedley, D., Krawitz, P., Robinson, P.N.: Jannovar: a Java library for exome annotation. Hum Mutat **35**(5), 548–555 (2014)
